# Transcriptomic analysis of two Chinese wheat landraces with contrasting Fusarium head blight resistance reveals miRNA-mediated defense mechanisms

**DOI:** 10.3389/fpls.2025.1537605

**Published:** 2025-02-28

**Authors:** Lijuan Wu, Junqiang Wang, Shian Shen, Zaijun Yang, Xinkun Hu

**Affiliations:** ^1^ Institute of Ecology, China West Normal University, Nanchong, Sichuan, China; ^2^ College of Agronomy, Sichuan Agricultural University, Chengdu, Sichuan, China; ^3^ College of Life Science, China West Normal University, Nanchong, Sichuan, China

**Keywords:** Sichuan wheat landraces, Fusarium head blight, lncRNAs, circRNAs, miRNAs, RNA sequencing

## Abstract

**Introduction:**

Fusarium head blight (FHB), caused primarily by *Fusarium graminearum* (*Fg*), poses a significant threat to wheat production. It is necessary to deeply understand the molecular mechanisms underlying FHB resistance in wheat breeding.

**Methods:**

In this study, the transcriptomic responses of two Chinese wheat landraces—Wuyangmai (WY, resistant) and Chinese Spring (CS, susceptible)—to *F. graminearum* infection were examined using RNA sequencing (RNA-seq). Differential expression of mRNAs, long non-coding RNAs (lncRNAs), circular RNAs (circRNAs), and microRNAs (miRNAs) was analyzed at 3 and 5 days post-*Fg* inoculation (dpi).

**Results:**

The results showed that WY exhibited a targeted miRNA response, primarily modulating defense-related pathways such as glutathione metabolism and phenylpropanoid biosynthesis, which are crucial for oxidative stress regulation and pathogen defense response. In contrast, CS displayed a broader transcriptional response, largely linked to general metabolic processes rather than immune activation. Notably, the up-regulation of genes involved in oxidative stress and immune defense in WY confirmed its enhanced resistance to FHB. The integrated analysis of miRNA-mRNA interactions highlighted miRNAs as central regulators of defense mechanisms in WY, particularly at later stages of infection. These miRNAs targeted genes involved in immune responses, while lncRNAs and circRNAs played a more limited role in the regulation of defense responses. The GO and KEGG pathway enrichment analyses further revealed that WY enriched for plant-pathogen interaction and secondary metabolite biosynthesis pathways, which are crucial for pathogen resistance. In contrast, CS prioritized metabolic homeostasis, suggesting a less effective defense strategy.

**Discussion:**

Overall, this study underscores the critical role of miRNA-mediated regulation in FHB resistance in WY. These insights into miRNA-mediated regulatory mechanisms provide a molecular basis for breeding FHB-resistant wheat varieties and highlight miRNA-mRNA interactions as promising targets for enhancing disease resilience.

## 1 Introduction

As one of the vital food crops, wheat (*Triticum aestivum* L.) supplies approximately 20% of the caloric intake for the global population (Ma et al., 2020). Wheat Fusarium head blight (FHB), destructive fungal disease primarily caused by *F. graminearum* (*Fg*), leads to a significant yield losses and quality deteriorates during epidemic years (Zhu et al., 2019). Additionally, FHB contaminates grain with harmful mycotoxins, including nivalenol and deoxynivalenol (DON), which seriously endanger the health of humans and livestock (Chen et al., 2019; Hu et al., 2023). Recent climate changes and wheat farming practices have increased the frequency and severity of FHB outbreaks (Ma et al., 2020). Identification and utilization of resistant germplasms in breeding programs are one of the most sustainable and economical approach to manage FHB.

FHB resistance is a complex quantitative trait governed by multiple genes and influenced by genotype-environment interactions (Zhang et al., 2021). Approximately 500 quantitative trait loci (QTLs) related to FHB resistance were identified from wheat and its relatives, and distributed across all 21 wheat chromosomes (Buerstmayr et al., 2009, Buerstmayr et al., 2020). However, most of these QTLs have minor effects and limited breeding value in addition to seven widely recognized major resistance genes (*Fhb1* to *Fhb7*). Recently, two additional major QTLs, *Fhb8* and *Fhb9*, were identified and associated with specific resistance types (Wang et al., 2024a; Zhang et al., 2024).

Despite these advances, only *Fhb1* and *Fhb7* have been cloned using map-based techniques and have shown significant resistance. *Fhb1* confers resistance through a histidine-rich calcium-binding protein (His or TaHRC), while the exact mechanism remains unclear (Su et al., 2019; Li et al., 2019a). *Fhb7*, derived from *Thinopyrum* species, encodes a glutathione-S-transferase (GST) that detoxifies trichothecene toxins (Wang et al., 2020a). However, wheat varieties incorporated with *Fhb7* are not yet widely available, and those with *Fhb1* displayed a moderate resistance, likely due to interactions with parent genotype (Li et al., 2019b). FHB resistance is generally inherited quantitatively and affected by multiple minor genes (Bai and Shaner, 2004). Other resistance genes, such as *TaFROG* and *TaABCC3*, have been identified and characterized for their roles in response to DON and *F. graminearum* (Perochon et al., 2015; Walter et al., 2015). To effectively combat FHB in wheat effectively, more effort is still needed to identify new resistant genes.

Transcriptome analysis is a powerful tool for studying FHB resistance mechanisms, offering insights into wheat-pathogen interactions. Previous studies utilized microarray techniques to analyze transcriptomic responses (Bernardo et al., 2007), while recent ones have applied RNA-seq to reveal new pathways and defense-related genes (Xiao et al., 2013; Brauer et al., 2019; Pan et al., 2018). Small RNAs, particularly miRNAs, have been found to regulate the response of wheat to *F. graminearum*, either by silencing fungal genes or modulating host defense pathways (MaChado et al., 2018; Jin et al., 2020; Biselli et al., 2018). However, little is known about the role of other non-coding RNAs, such as long non-coding RNAs (lncRNAs) and circular RNAs (circRNAs), in response to FHB resistance.

Chinese wheat landraces are gene pools for FHB-resistant. Some of the well-known resistant resources including Wangshuibai, Sanyuehuang, Shuilizhan, and Wuyangmai (WY), originated in China (Wan et al., 1997). WY, in particular, is an FHB-resistant landrace from Yibin city in Sichuan, China. However, the loci against FHB in WY have not yet been genetically identified, and the mechanisms underlying FHB resistance remain unknown. In this study, RNA-seq analysis was performed on WY (an FHB-resistant genotype) and Chinese Spring (CS, an FHB-susceptible genotype) in response to *F. graminearum* at 3 and 5 dpi to identify lncRNAs, circRNAs, miRNAs, and mRNAs involved in host-pathogen interactions. Differentially expressed miRNAs and mRNAs were used as central elements to target other differentially expressed RNAs, including lncRNAs and host genes associated with circRNAs. Additionally, differential expression analysis, along with Gene Ontology (GO) term and Kyoto Encyclopedia of Genes and Genomes (KEGG) pathway enrichment analyses, was conducted on significantly up-regulated genes in CS and WY at different time points. Finally, the regulatory relationships between differentially expressed miRNAs and mRNAs were characterized, leading to the construction of a miRNA-mRNA targeting network map. The results provide valuable information for gaining a deeper understanding the FHB resistance differences of the two Sichuan wheat landraces, WY and CS.

## 2 Materials and methods

### 2.1 Plant materials and cultivation conditions

The two Sichuan wheat landraces, WY and CS, which exhibit high resistance (HR) and high susceptibility (HS) to FHB, respectively (Wan et al., 1997), were used in this study. The two landraces were provided by the Triticeae research Institute of Sichuan Agricultural University of China, and showed similar plant heights, spikes, heading and flowering dates, minimizing the potential discrepancies due to variations in plant architecture and development periods. The experiment was conducted at the breeding field of China West Normal University (Nanchong, Sichuan Province, China; 30°48′N, 106°05′E) during the 2022-2023 growing season. A randomized block design was implemented, with each genotype sown in a plot containing ten rows. Each row was 1.5 meters long, with 20 seedlings spaced 0.3 meters apart. Field cultivation and fertilization practice were the same as local wheat cultivation standards, and no fungicide was applied.

### 2.2 Macroconidia preparation and *F. graminearum* inoculation

A highly virulent and 15-acetyldeoxynivalenol (15ADON) producing isolate of *F. graminearum*, F0609, supplied by the Jiangsu Academy of Agricultural Science (Nanjing, Jiangsu Province, China), was used to infect the wheat plants. To prepare the macroconidia, isolate F0609 was first cultured on potato dextrose agar (PDA) medium at room temperature for 10 days under fluorescent-UV lights. A small piece of mycelium was then transferred to a mung bean medium and shaken at 180 rpm at 28°C for 4 days. The mycelia were filtered with double-layered medical gauze, and the macroconidia concentration was measured by a blood cell counting plate under a microscope. The macroconidia suspension was then diluted to a concentration of 1 × 10^5^ spores/mL.

At mid-anthesis, 10 µL of the *F. graminearum* macroconidial spore suspension (1 × 10^5^ spores/mL) was point-inoculated into the two basal florets of fully developed spikelet between the lemma and palea using a micropipette (Hu et al., 2019). Each wheat genotype was inoculated with three biological replicates, each consisting of 18 spikes. The inoculated spikes were then covered with a transparent plastic bag to maintain humidity and sprayed with sterile water twice daily until harvest. Spikelets were harvested at 0 (sterile water mock inoculation control), 3, and 5 dpi, with three biological replicates for each time point. Those samples collected from CS and WY at 0, 3 and 5 dpi were designated as CS-0, CS-3, CS-5, WY-0, WY-3, and WY-5, respectively. A total of nine samples were collected for each genotype.

### 2.3 RNA extraction, library construction and sequencing

Total RNA of each sample was extracted with the TRIzol reagent (Invitrogen, CA, USA), and the residual DNA was removed using RNase-free DNase I (Invitrogen, CA, USA) following the manufacturer’s instructions. The integrity and quality of the RNA were preliminarily assessed with 1.5% formaldehyde denaturing agarose gel electrophoresis. The concentration and purity of RNA were measured by a NanoDrop 2000 Spectrophotometer (Thermo Fisher Scientific, MA, USA). The RNA integrity was further determined by using the RNA 6000 Nano Assay Kit on the Agilent Bioanalyzer 2100 System (Agilent, CA, USA).

For cDNA library construction, 1.5 μg of RNA per sample was used for rRNA removal with the Ribo-Zero rRNA Removal Kit (Epicentre, Madison, WI, USA). Sequencing libraries were then prepared with the NEBNext^®^ Ultra™ Directional RNA Library Prep Kit for Illumina^®^ (NEB, MA, USA) following the manufacturer’s guidelines, with index codes included for sample identification. For small RNA (sRNA) library construction, 2.5 μg of RNA per sample was used. Sequencing libraries were generated with the NEBNext^®^ Multiplex Small RNA Library Prep Set for Illumina^®^ (NEB, MA, USA) based on the manufacturer’s instructions, and index codes were also added for sample identification (Zhang et al., 2020). The quality of all libraries was assessed using the Agilent Bioanalyzer 2100. Paired-end sequencing (PE150-bp) was performed on the Illumina NovaSeq 6000 platform (Illumina, San Diego, CA, USA) by Biomarker Technologies (Qingdao, China).

### 2.4 Data processing

The raw data (raw reads) were filtered with in-house Perl scripts to remove low-quality reads and sequences containing adapters or poly-N reads. Clean data (clean reads) were collected, and analyzed for the Q20, Q30, GC content, and sequence duplication levels. For sRNA-seq data, sequences ranging from 15 to 35 nucleotides (nt) in length were retained after trimming. All downstream analyses were conducted with high-quality clean data. The clean reads were then mapped to the CS reference genome IWGSC_RefSeq_v2.1 (IWGSC; Alaux et al., 2023) using HISAT2 software version 2.2.1 (Kim et al., 2019). Only reads with a perfect match or a single mismatch were further analyzed and annotated based on the reference genome.

### 2.5 Identification of mRNA, lncRNA, circRNA, and miRNA

For mRNA and lncRNA identification, the transcriptome was assembled using StringTie (v2.2.0) (Kovaka et al., 2019) based on the reads mapped to the wheat CS reference genome (IWGSC_RefSeq_v2.1). The GffCompare program (v0.12.6) (Pertea and Pertea, 2020) was used to annotate the assembled transcripts. Unknown transcripts were screened as potential lncRNAs. Four computational tools—CPC, CNCI, Pfam, and CPAT—were combined to distinguish non-protein-coding RNA (ncRNA) candidates from the unknown transcripts. Putative protein-coding RNAs were filtered out using thresholds for minimum transcript length and exon number. LncRNA candidates were selected based on transcript lengths greater than 200 nucleotides (nt) and more than 2 exons, and further validated with the four computational tools mentioned above. Those tools effectively differentiate protein-coding genes from non-coding ones and classify various lncRNA types, such as lincRNAs, intronic lncRNAs, antisense, and sense lncRNAs (Zhang et al., 2020).

The circRNA identification tool “CIRI” (CircRNA Identifier) (Gao et al., 2015) was used to detect circRNAs from the transcriptome data. To collect sufficient information for circRNA identification and characterization, the SAM files were scanned twice with CIRI. The identified circRNAs were then output with annotation information. Target miRNAs of the circRNAs and the target genes of miRNAs were predicted using miRanda (Doron et al., 2008) and RNAhybrid for animals (Rehmsmeier et al., 2004), and TargetFinder for plants (Bo and Wang, 2005). During the predictions, FASTA sequences of these circRNAs and miRNAs were used as input files.

The clean reads were aligned against four bioinformatic databases, Silva, GtRNAdb, Rfam, and Repbase with Bowtie software (Langmead and Salzberg, 2012) to identify candidate miRNA. The alignments allowed for length variations at both the 5′ and 3′ ends, as well as one internal mismatch. Common RNA families, including rRNA, tRNA, snRNA, snoRNA, and other ncRNAs, along with repeats, were filtered out. The remaining reads were then subject to miRNA identification through comparison with the wheat reference genome. The known and novel miRNAs were detected with the miRBase v22 database (Kozomara et al., 2019) and miRDeep2 module (Friedländer et al., 2012), respectively.

### 2.6 Differential expression analysis

The expression levels of coding genes and lncRNAs in each sample were estimated with fragments per kilobase of transcript per million fragments mapped (FPKM) values (Trapnell et al., 2010). The expression of circRNAs was determined based on the number of junction reads identified by the CIRI tool. For miRNAs, expression levels of each sample were estimated through the following steps: (1) sRNAs were mapped back to the precursor sequence, and (2) the read count for each miRNA was obtained from the mapping results. The expression levels of miRNAs and circRNAs in each sample were calculated using transcripts per million (TPM) (Fahlgren et al., 2007). Differential expression analysis among three time points (0, 3, and 5 dpi) of two genotypes (WY and CS) was conducted with DESeq2 R package (v1.10.1) (Love et al., 2014). Differentially expressed genes (DEGs) were identified using an adjusted *p*-value (calculated using the Benjamini and Hochberg method) ≤ 0.01 to control the false discovery rate (FDR) and a |log_2_ (Fold change (FC))| > 1. Venny 2.1 (Oliveros, 2015) was then used for Venn diagram analysis, and SRplot web server (Tang et al., 2023; available at http://www.bioinformatics.com.cn/SRplot) was used for heatmap drawing.

### 2.7 Analysis of competing endogenous RNA

Candidate ceRNA relationship pairs were identified based on miRNA targeting relationships. Using the predicted miRNA-mRNA, lncRNA-miRNA, and circRNA-miRNA interaction pairs, groups of lncRNA-miRNA-mRNA or circRNA-miRNA-mRNA that shared the same miRNAs were collected. The screening criteria for ceRNAs were as follows: (1) The number of shared miRNAs among ceRNAs must be greater than or equal to 5; (2) The hypergeometric test *p*-value must be < 0.01, and the corrected FDR value must be < 0.01; (3) mRNAs and lncRNA/circRNA interaction pairs with a co-expression correlation (r > 0.9) were further selected for ceRNA network construction.

### 2.8 Gene functional annotation and GO and KEGG enrichment analysis

Gene function annotation was performed using multiple databases, including Nr (Non-redundant protein), Nt (Non-redundant nucleotide), Pfam (Protein family), KOG/COG (Clusters of Orthologous Groups of proteins), Swiss-Prot (a manually annotated and non-redundant protein sequence database), KEGG, and GO. GO enrichment analysis of DEGs was conducted with the GOseq R package, based on Wallenius’ non-central hypergeometric distribution (Young et al., 2010), which adjusts for gene length bias in DEGs. The enrichment analysis of DEGs in KEGG pathways was performed using KOBAS software (version 2.0) (Xie et al., 2011).

### 2.9 Real-time quantitative PCR validation

For RT-qPCR analysis, cDNA of all RNA samples (those used for Illumina RNA-sequencing) was synthesized with the PrimeScript RT reagent Kit (TaKaRa, Dalian, China) according to the manufacturer’s protocol. The reaction volume was 20 μL including random hexamers and 1 μg of each RNA sample. Two wheat genes—glyceraldehyde-3-phosphate dehydrogenase (GAPDH, TraesCS7A02G313100) and indole-3 acetaldehyde oxidase (IAAOx, TraesCS2A02G246300) (Hu et al., 2019)—were used as reference genes to normalize gene expression in the two wheat genotypes. Primers (**Dataset S1**) were designed using the PrimerQuest™ Tool from Integrated DNA Technologies (IDT) and synthesized by Sangon Biotech (Shanghai, China). For each reaction, 1 μL of 10×-diluted cDNA was added to a 10 μL reaction volume using the SYBR Green PCR kit (TaKaRa, Dalian, China). RT-qPCR was carried out in an ABI7500 Real-Time PCR System (Applied Biosystems, CA, USA). The cycling conditions were 5 minutes at 95°C, followed by 40 cycles of 30 seconds at 95°C, 30 seconds at the melting temperature (as indicated in **Dataset S1**), and 30 seconds at 72°C. The melting curve analysis was conducted from 55°C to 95°C, with readings taken every 1°C and held for 5 seconds. Four technical replicates were performed for each sample. The relative expression levels were calculated using the 2^−ΔΔCt^ method (Livak and Schmittgen, 2001).

## 3 Results

### 3.1 Sequencing and identification of RNAs

After sequencing of the 18 samples, the raw data were filtered to obtain clean data, resulting in a total of 1,035,915,752 reads and 309.95 Gb of clean data. Each sample yielded approximately 15.95 Gb of clean data, with Q20 and Q30 base percentages exceeding 97.02% and 92.05%, respectively. The GC content ranged from 47.39% to 50.51% (**Dataset S2**). These metrics indicate that the quality of RNA sequencing in this study was high and suitable for further analysis. The clean reads were mapped to the reference wheat genome sequences (IWGSC_RefSeq_v2.1) of Chinese Spring (Alaux et al., 2023). After identification of RNAs, a total of 15,765 lncRNAs, 286,397 genes, 621 circRNAs, and 3,063 miRNAs were detected.

### 3.2 Differential expression analysis of mRNA, lncRNA, circRNA, and miRNA

**Figure 1:**
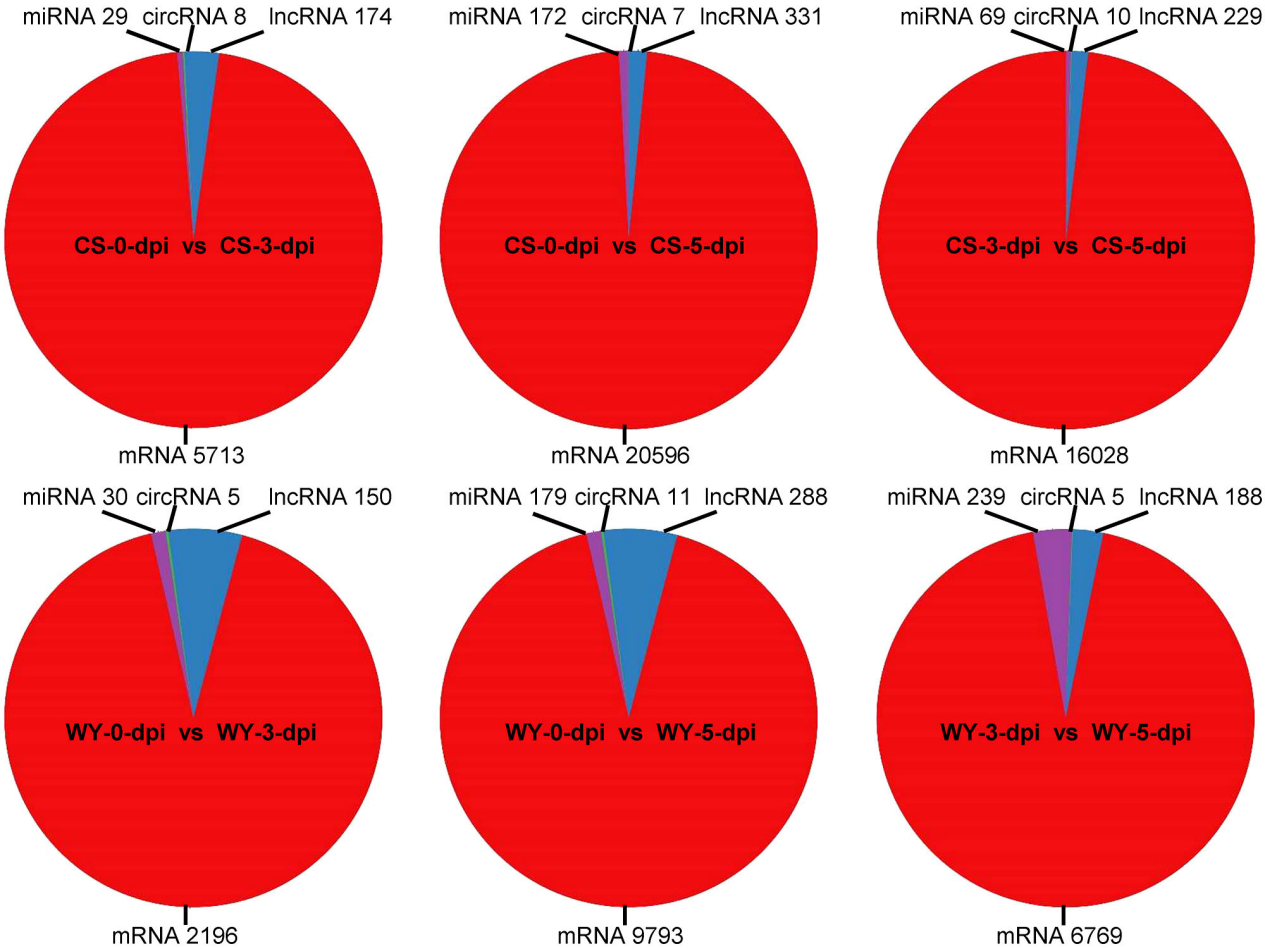
Statistics of differentially expressed RNAs across various comparisons. The 0, 3, and 5-dpi represent samples collected at 0, 3, and 5 days post *F. graminearum* inoculation (dpi), respectively. CS and WY refer to Chinese Spring and Wuyangmai, respectively. LncRNA, long non-coding RNA; miRNA, microRNA; circRNA, circular RNA.

**Figure 2:**
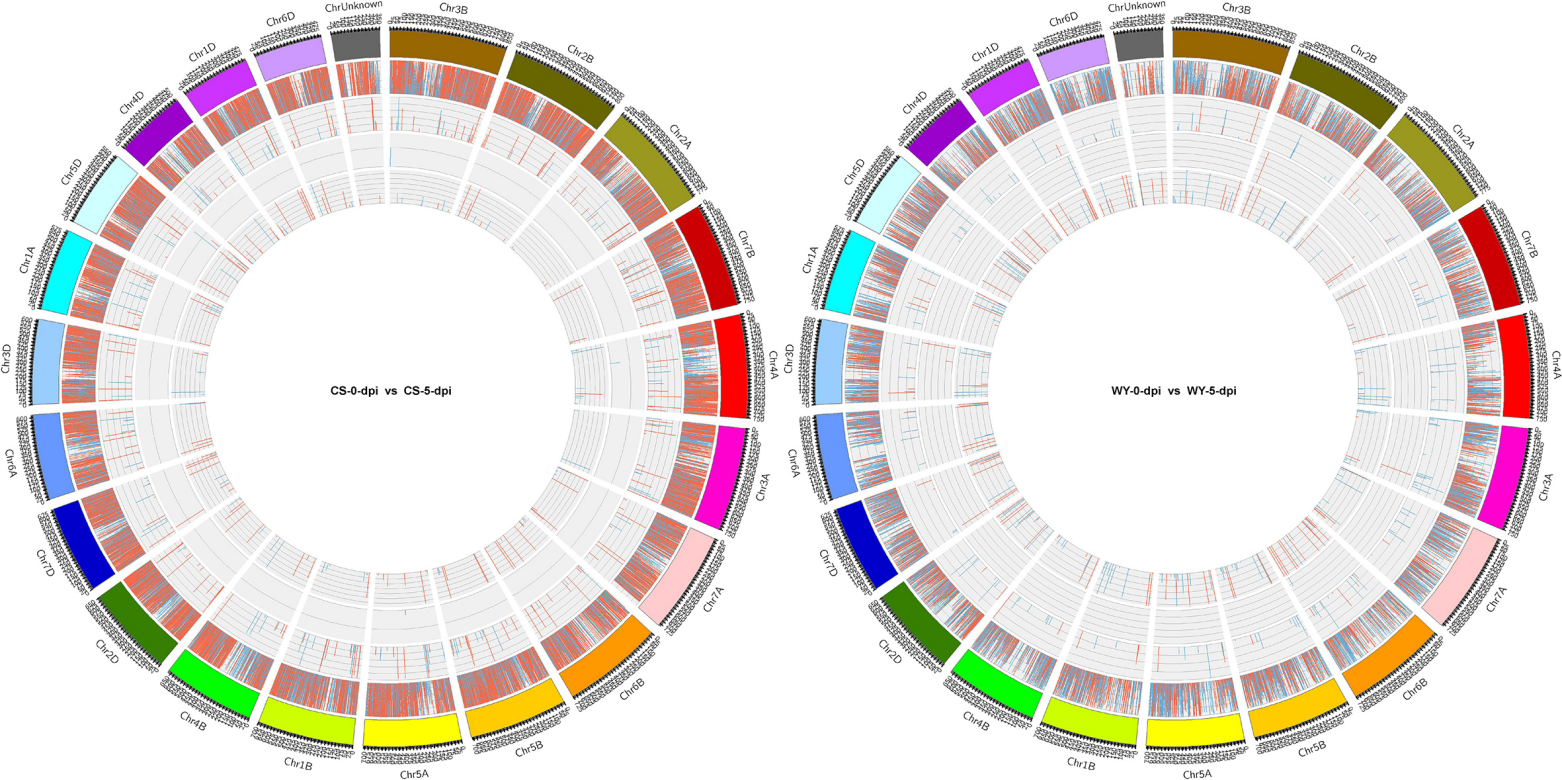
Expression profiles of differentially expressed RNAs at 5 dpi with *F. graminearum*. The outermost ring shows chromosome information, followed by mRNA (gene), lncRNA, circRNA, and miRNA. For each group of differentially expressed RNAs, red, blue, and the height indicate up-regulation, down-regulation, and significance [-log_10_ (FDR)], respectively.

To identify differentially expressed RNAs, pairwise comparisons were conducted between time points for the two wheat genotypes. In CS, the numbers of differentially expressed mRNAs, lncRNAs, circRNAs, and miRNAs for 0 *vs*. 3-dpi, 0 *vs*. 5-dpi, and 3 *vs*. 5-dpi, were 5,713, 174, 8, and 29; 20,596, 331, 7, and 172; and 16,028, 229, 10, and 69, respectively. In WY, the numbers of differentially expressed mRNAs, lncRNAs, circRNAs, and miRNAs for 0 *vs*. 3-dpi, 0 *vs*. 5-dpi, and 3 *vs*. 5-dpi were 2,196, 150, 5, and 30; 9,793, 288, 11, and 179; and 6,769, 188, 5, and 239, respectively (**Figure 1**). The expression profiles of those differentially expressed RNAs were visualized using circos maps, with the height of the profiles representing significance (-log_10_ (FDR)). The results showed that the *F. graminearum* invasion had a significant impact on the expression of mRNA and miRNA, and had moderate to low effects on the expression of lncRNAs and circRNAs, respectively (**Figure 2**) (**Supplementary Figure 1**).

Analysis of the differentially expressed RNAs showed that the numbers of differentially expressed genes in CS were 2.60, 2.10, and 2.37 times greater than those in WY for the comparison time points of 0 *vs*. 3-dpi, 0 *vs*. 5-dpi, and 3 *vs*. 5-dpi, respectively. The differentially expressed lncRNAs in CS were slightly higher than those in WY, with fold changes of 1.16, 1.15, and 1.22 for the above-mentioned time points. However, the numbers of differentially expressed circRNAs were very low in both CS and WY, ranging from 5 to 11. Interestingly, the number of differentially expressed miRNAs in WY was slightly higher than that in CS for the comparison time points of 0 *vs*. 3 dpi and 0 *vs*. 5 dpi, and 3.46 times higher for the comparison time points of 3 *vs*. 5 dpi (**Table 1**).

**Table 1:** Comparative analysis of differentially expressed RNAs between Chinese Spring (CS) and Wuyangmai (WY).

RNA	0 *vs*. 3-dpi			0 *vs*. 5-dpi			3 *vs*. 5-dpi		
	CS	WY	CS/WY	CS	WY	CS/WY	CS	WY	CS/WY
mRNA	5713	2196	2.60	20596	9793	2.10	16028	6769	2.37
lncRNA	174	150	1.16	331	288	1.15	229	188	1.22
circRNA	8	5	1.6	7	11	0.66	10	5	2.00
miRNA	29	30	0.97	172	179	0.96	69	239	0.29

### 3.3 Integrated analysis of differentially expressed RNA targeting relationships

Differentially expressed lncRNAs, circRNAs, miRNAs, and mRNAs were analyzed by taking each RNA type as the center and examining the targeted relationships involving other differentially expressed RNAs (including host genes corresponding to circRNAs). First, an integrated analysis was conducted using differentially expressed lncRNAs (DE_lncRNAs) as the center. In the comparisons of 0 *vs*. 3-dpi, 0 *vs*. 5-dpi, and 3 *vs*. 5-dpi of CS, the Venn diagram revealed that 0 (1), 11 (14), and 2 (4) lncRNAs, respectively, were located at the intersection of the three data sets: DE_lncRNA, DE_Cis.mRNA_Target lncRNA (or DE_Trans.mRNA_Target lncRNA), and DE_miRNA_Target lncRNA. Similarly, for the pairwise comparisons of the three time points in WY, the Venn diagram indicated that 0 (0), 1 (7), and 1 (4) lncRNAs were located at the intersection of the same three data sets, respectively (**Supplementary Figures 2A, B**). When circRNAs were taken as the center for the targeting relationship analysis, no circRNAs were found at the intersection of the three data sets: DE_circRNA, DE_Hostgene_circRNA, and DE_miRNA_Target circRNA, for any of the pairwise comparisons among the three time points in CS or WY (**Supplementary Figure 3**).

**Figure 3:**
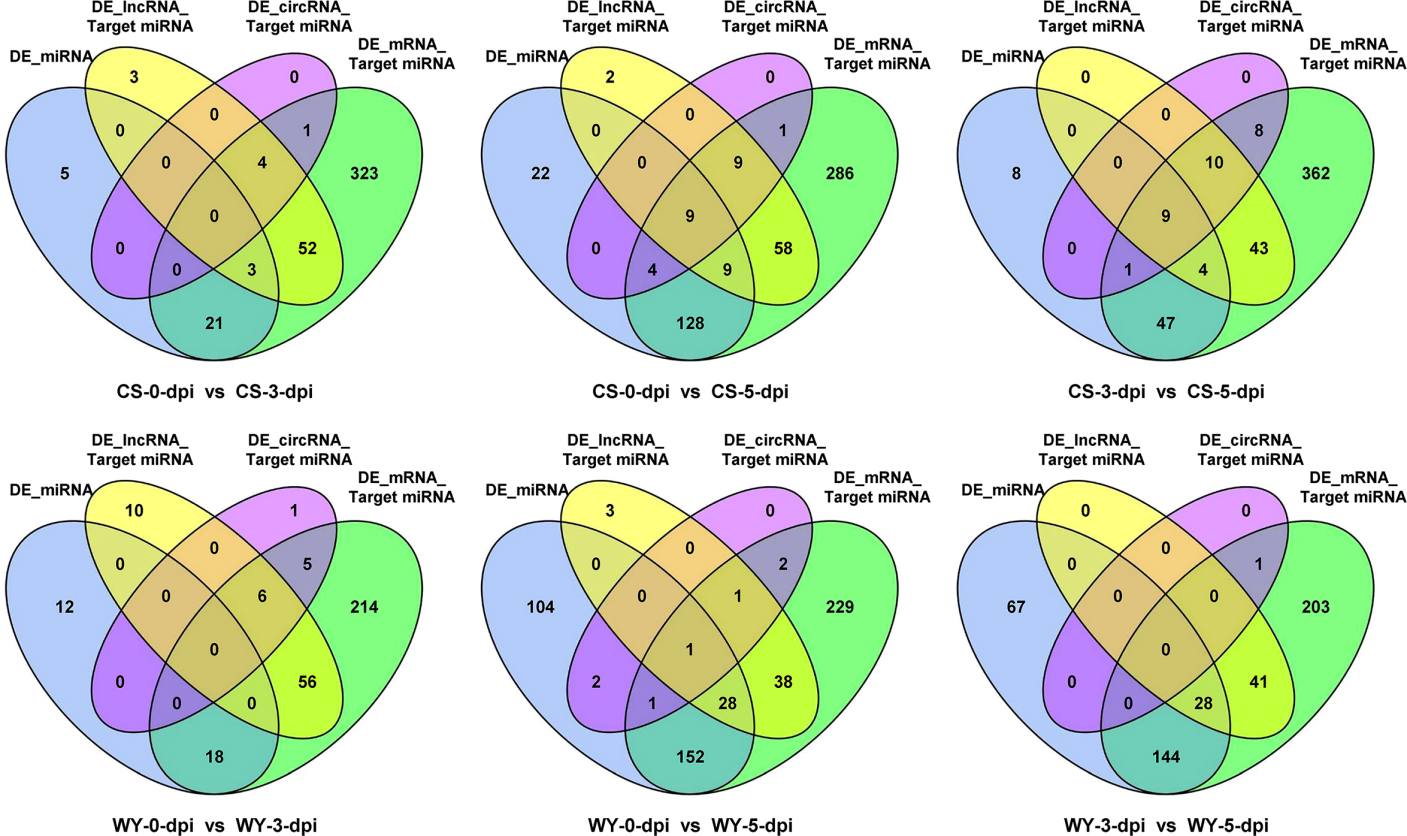
Interaction of differentially expressed miRNAs with all miRNAs targeted by differentially expressed mRNAs, lncRNAs, and circRNAs. Here, DE_miRNA represents differentially expressed miRNAs; DE_mRNA_Target miRNA, DE_lncRNA_Target miRNA, and DE_circRNA_Target miRNA represent miRNAs targeted by differentially expressed genes, lncRNAs, and circRNAs, respectively.

Subsequently, an integrated analysis of targeting relationships was conducted using differentially expressed miRNAs (DE_miRNA) as the center. In the pairwise comparisons of the three time points in CS, the Venn diagram indicated that 0 (3), 4 (9), and 1 (4) miRNA were present at the intersection of the three data sets: DE_miRNA, DE_circRNA_Target miRNA (or DE_lncRNA_Target miRNA), and DE_mRNA_Target miRNA, respectively. Similarly, for the pairwise comparisons of the three time points in WY, the Venn diagram showed that 0 (0), 1 (28), and 0 (28) miRNAs were located at the intersections of the same data sets, respectively (**Figure 3**). Additionally, the analysis revealed that the primary targeting relationships existed between the DE_miRNA and DE_mRNA_Target miRNA data sets. Specifically, 24 (18), 150 (182), and 61 (172) miRNAs were identified at their intersection in the pairwise comparisons of the three CS (or WY) time points, respectively (**Figure 3**).

Finally, differentially expressed mRNAs (DE_mRNA) were used as the center for analyzing targeting relationships. The Venn diagram showed that the primary targeting relationships occurred between the DE_mRNA data set and either DE_lncRNA_Target mRNA or DE_miRNA_Target mRNA. In the pairwise comparisons of the three CS time points, 39 (1,241), 283 (7,027), and 166 (5,608) mRNAs were detected at the intersection of DE_mRNA with DE_lncRNA_Target Cis.mRNA (or DE_lncRNA_Target Trans.mRNA), respectively. Similarly, for the pairwise comparisons of the three WY time points, 12 (167), 42 (1,551), and 36 (1,358) mRNAs were found at the corresponding intersections of the above two data sets (**Supplementary Figures 4A, B**). When considering the targeting relationships between DE_mRNA and DE_miRNA_Target mRNA, 72 (23), 2,152 (1,239), and 1,179 (783) mRNAs were identified at their intersections in the pairwise comparisons of the three CS (or WY) time points, respectively (**Supplementary Figures 4A, B**).

### 3.4 Identification of ceRNA

mRNAs, lncRNAs, and circRNAs can function as competing endogenous RNAs (ceRNAs) in a ceRNA network, where they regulate expression levels of each other by competitively binding to the same miRNA response elements (MREs) (Salmena et al., 2011). Based on the screening criteria for ceRNAs, co-expression analysis identified 98 circRNA-miRNA-mRNA relationship pairs and 49 lncRNA-miRNA-mRNA pairs (**Dataset S3**). However, no differentially expressed ceRNA relationship pairs were identified using a one-step nearest-neighbor network analysis, when each group of differentially expressed RNAs was extracted from the ceRNA relationship pairs.

### 3.5 Differential expression analysis for up-regulated genes

**Figure 4:**
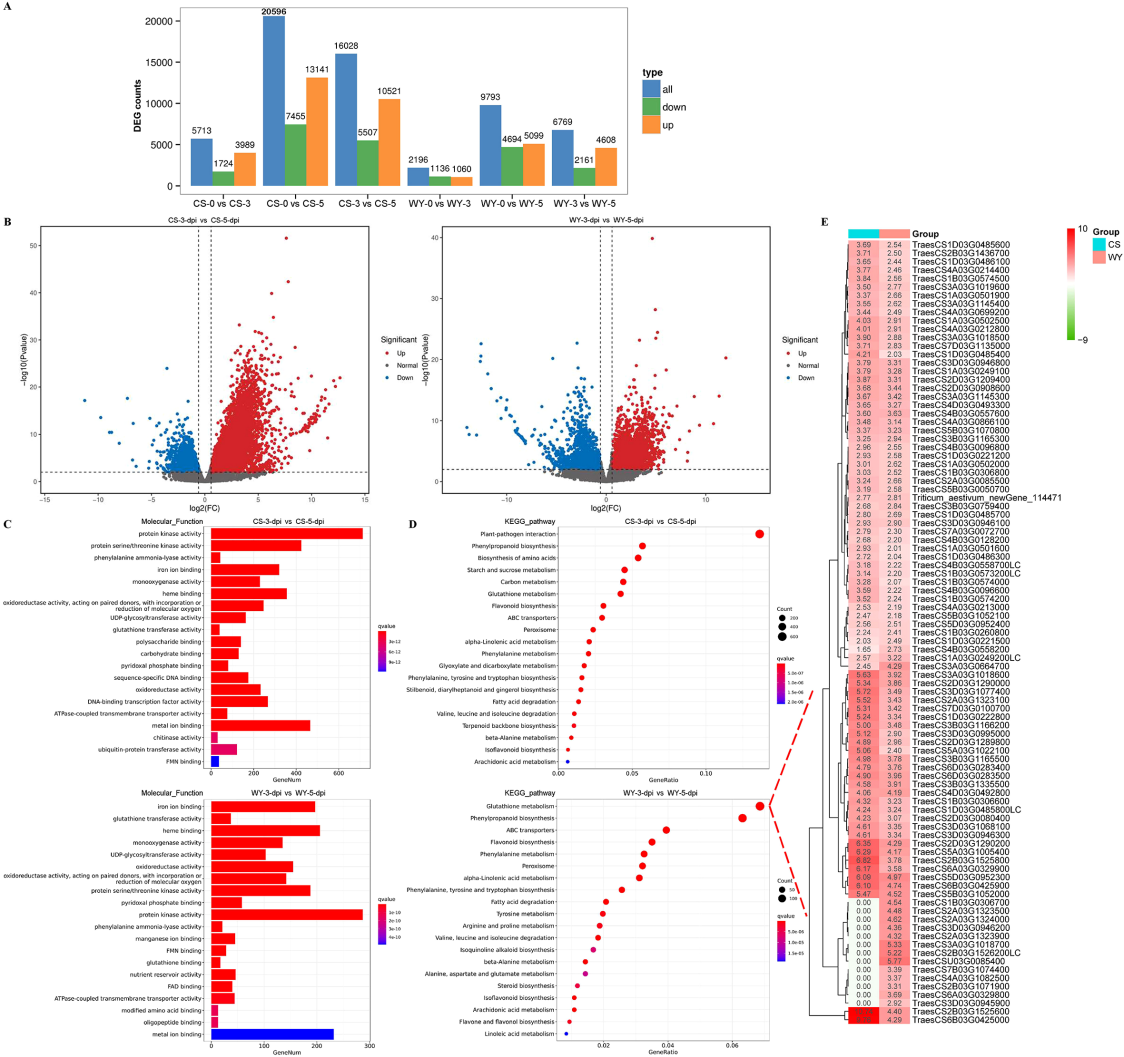
Differential expression analysis for up-regulated genes. **(A)** Statistical analysis for all genes. **(B)** Volcano plot of DEGs obtained in the comparison of 3 *vs*. 5-dpi. **(C)** GO term and **(D)** KEGG pathway enrichment analysis for significantly up-regulated DEGs. **(E)** Gene expression heatmap of glutathione metabolism pathway.

Differential gene expression analysis revealed that in CS, there were 3,989, 13,141, and 10,521 up-regulated DEGs, and 1,724, 7,455, and 5,507 down-regulated DEGs for 0 *vs*. 3-dpi, 0 *vs*. 5-dpi, and 3 *vs*. 5-dpi comparisons, respectively. The number of up-regulated DEGs was consistently much greater than that of down-regulated DEGs across all three comparisons. In WY, there were 1,060, 5,099, and 4,608 up-regulated DEGs, and 1,136, 4,694, and 2,161 down-regulated DEGs for the same comparisons. The number of up-regulated DEGs was higher than down-regulated DEGs only in the 3 *vs*. 5-dpi comparison, while for the 0 *vs*. 3-dpi and 0 *vs*. 5-dpi comparisons, the up-regulated DEGs were slightly fewer or greater than the down-regulated ones, respectively (**Figure 4A**). Additionally, the expression levels and significance of up-regulated DEGs in CS were consistently much higher than those in WY across all three comparisons (**Figure 4B**) (**Supplementary Figures 5A, 6A**).

GO enrichment analysis of up-regulated DEGs revealed distinct defense responses between the FHB-resistant wheat CS and the susceptible wheat WY. For the 0 *vs*. 3-dpi comparison, two of the top five GO terms differed between CS and WY. In CS, the top terms were “phenylalanine ammonia-lyase activity” and “heme binding”, while in WY, they were “hydrolase activity, hydrolyzing O-glycosyl compounds” and “polysaccharide binding” (**Supplementary Figure 5B**). In the 0 *vs*. 5-dpi comparison, CS showed enrichment for “glutathione transferase activity” and “iron ion binding”, while WY had “ATPase-coupled transmembrane transporter activity” and “ATPase activity” (**Supplementary Figure 6B**). In the 3 *vs*. 5-dpi comparison, three of the top five GO terms were different: CS showed “protein kinase activity”, “protein serine/threonine kinase activity”, and “phenylalanine ammonia-lyase activity”, while WY showed “glutathione transferase activity”, “heme binding”, and “UDP-glycosyltransferase activity” (**Figure 4C**).

KEGG pathway enrichment of up-regulated DEGs also highlighted distinct defense responses between CS and WY. In the 0 *vs*. 3-dpi comparison, only one of the top five KEGG pathways was different: “biosynthesis of amino acids” (ko01230) in CS, compared to “plant hormone signal transduction” (ko04075) in WY, although the enrichment of the top five pathways in WY was not significant. In the 0 *vs*. 5-dpi comparison, two of the top five pathways differed: “phenylpropanoid biosynthesis” (ko00940) and “carbon metabolism” (ko02010) in CS, and “glutathione metabolism” (ko00480) and “ABC transporters” (ko02010) in WY. For the 3 *vs*. 5-dpi comparison, four of the top five pathways were different between CS and WY: “plant-pathogen interaction” (ko04626), “biosynthesis of amino acids” (ko01230), “starch and sucrose metabolism” (ko00500), and “carbon metabolism” (ko01200) in CS, compared to “glutathione metabolism” (ko00480), “ABC transporters” (ko02010), “flavonoid biosynthesis” (ko00941), and “phenylalanine metabolism” (ko00360) in WY (**Figure 4D**) (**Supplementary Figures 5C**, **6C**).

Additionally, a log_2_ (FC) > 2 threshold was applied to filter up-regulated genes enriched in the “glutathione metabolism” pathway in the 3 *vs*. 5-dpi comparison of WY, resulting in the identification of 93 DEGs. The expression heatmap of these up-regulated DEGs showed that 80 DEGs were shared between CS and WY. Among these, two DEGs (TraesCS2B03G1525600 and TraesCS6B03G0425000) in CS had expression levels more than two times higher than in WY. Furthermore, the heatmap also showed that 13 DEGs were differentially expressed only in WY (**Figure 4E**).

### 3.6 Integrated analysis of the targeting relationship between miRNA and mRNA

Using small RNA and transcriptome sequencing data, DE_miRNAs and DEGs were identified in the two sample groups. The regulatory relationships between miRNAs and mRNAs were then explored, focusing on the negative regulatory effects of miRNAs on mRNAs. First, DE_miRNAs were used as screening criteria to identify the mRNAs regulated by these miRNAs, followed by an analysis of the pairs of DE_miRNAs and mRNAs with negative regulatory relationships (**Dataset S4**). Similarly, DEGs were used as screening criteria to identify miRNAs that regulate these DEGs, and DEG-miRNA pairs with negative regulatory relationships were analyzed (**Dataset S5**). The targeting relationships between miRNAs and mRNAs were visualized using Cytoscape software (v3.10.2). The top 20 DE_miRNAs (or top 18 for 0 *vs*. 3-dpi comparison in WY) most relevant to target gene regulation were selected to construct the miRNA-mRNA targeting relationship network map (**Dataset S6**).

**Figure 5:**
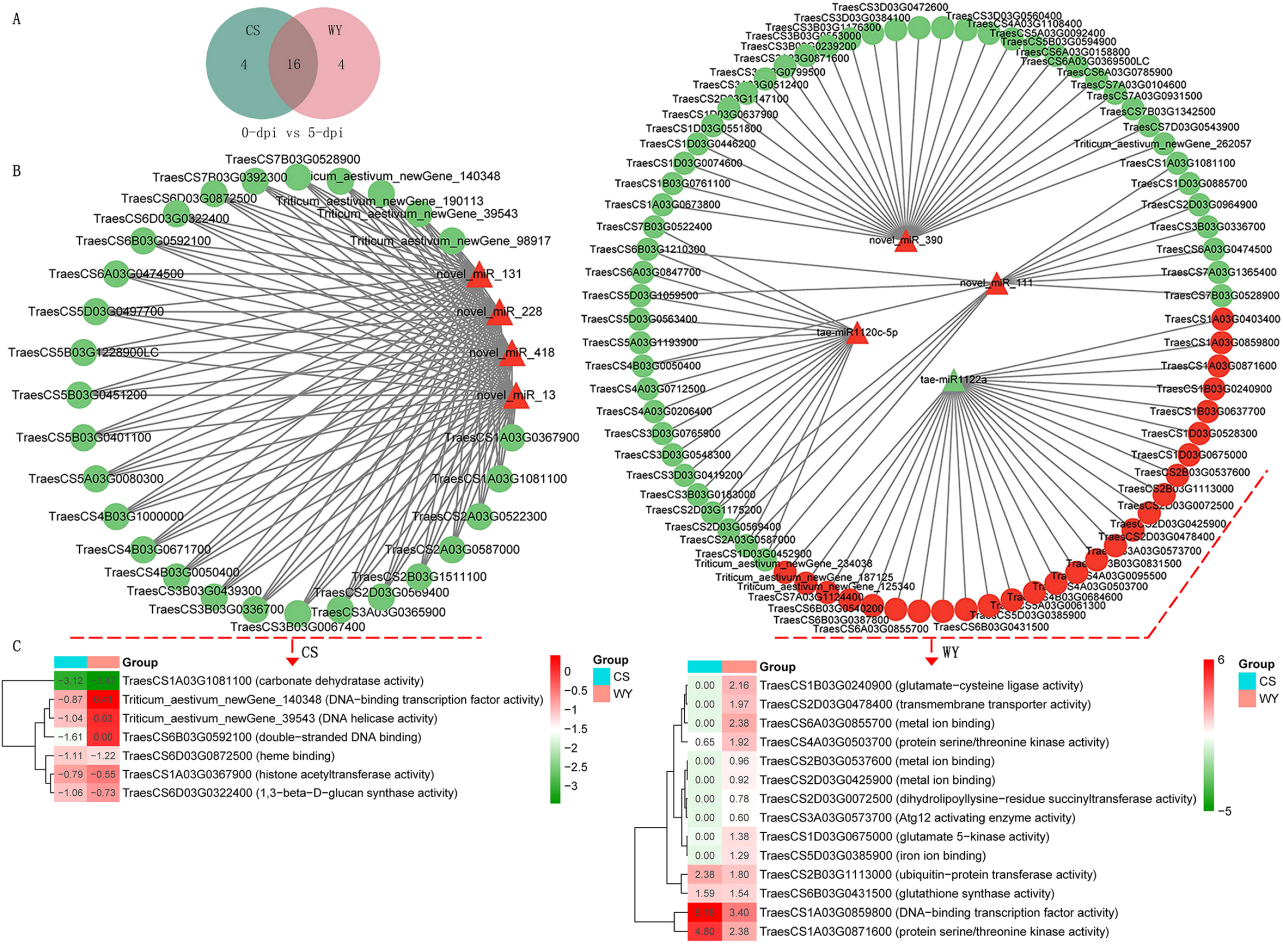
Targeting relationships between DE_miRNAs and DEGs. **(A)** Comparison of the top 20 DE_miRNAs most relevant to target gene regulation in CS and WY. **(B)** Targeting relationships between the DE_miRNAs specifically expressed in CS and WY and DEGs. **(C)** Expression heatmap of genes significantly down-regulated by novel_miR_228 and significantly up-regulated by tae-miR1122a with molecular function (MF) GO term. Heatmap represents log_2_ (FC) between *F. graminearum* infected and control.

The results showed that only two miRNAs were shared between CS and WY in the 0 *vs*. 3-dpi comparison, whereas 16 and 15 miRNAs were shared in the 0 *vs*. 5-dpi and 3 *vs*. 5-dpi comparisons, respectively (**Figure 5A**) (**Supplementary Figure 7A**). In the 0 *vs*. 3-dpi comparison, ten DE_miRNAs in CS and eight in WY specifically targeted DEGs with negative regulatory relationships, with each DE_miRNA targeting one to four DEGs (**Supplementary Figure 7B**). In the 0 *vs*. 5-dpi and 3 *vs*. 5-dpi comparisons, all DE_miRNAs specifically expressed in CS and WY targeted DEGs with negative regulatory relationships, with each DE_miRNA targeting 14 to 30 DEGs. Notably, novel_miR_228, which was up-regulated in CS, and tae-miR1122a, which was down-regulated in WY, were present in both comparisons. Novel_miR_228 down-regulated 28 and 23 DEGs in CS, while tae-miR1122a up-regulated 27 and 23 DEGs in WY, respectively (**Figure 5B**) (**Supplementary Figure 7B**).

GO analysis of these DEGs revealed that seven DEGs down-regulated by novel_miR_228 in CS had molecular function (MF) terms in the 0 *vs*. 5-dpi comparison, and nine DEGs in the 3 *vs*. 5-dpi comparison. Significant GO term enrichment analysis (using a *q*-value < 0.05 as the threshold) showed two DEGs, TraesCS1A03G0367900 (histone acetyltransferase activity; *q* = 1.52e-02) and TraesCS6B03G0592100 (double-stranded DNA binding; *q* = 1.67e-43), were significantly enriched in the 0 *vs*. 5-dpi comparison of CS, while six DEGs—TraesCS2A03G0587000 (protein kinase activity; *q* = 9.31e-06), TraesCS2D03G0569400 (protein kinase activity; *q* = 9.31e-06), TraesCS3D03G0550100 (hydrolase activity; *q* = 1.61e-02), TraesCS5A03G0080300 (microtubule binding; *q* = 1.12e-02), TraesCS7A03G0224800 (single-stranded DNA binding; *q* = 4.39e-04), and TraesCS7A03G0450300 (GTP binding; *q* = 4.47e-08)—were significantly enriched in the 3 *vs*. 5-dpi comparison of CS. The expression of these DEGs in CS was lower than in WY (**Figure 5C**) (**Supplementary Figure 7C**).

Likewise, GO analysis showed that 14 DEGs up-regulated by tae-miR1122a in WY had MF terms in both the 0 *vs*. 5-dpi and 3 *vs*. 5-dpi comparisons. Nine DEGs up-regulated by tae-miR1122a in WY were not differentially expressed in CS in the 0 *vs*. 5-dpi comparison, and three (TraesCS2B03G0537600, TraesCS2D03G0425900, and TraesCS6A03G0855700) were significantly enriched in the MF term for metal ion binding (*q* = 2.14e-03). In the 3 *vs*. 5-dpi comparison, nine DEGs up-regulated by tae-miR1122a in WY were not differentially expressed in CS, and four (TraesCS2B03G0537600, TraesCS2D03G0425900, TraesCS6A03G0855700, and TraesCS7D03G0982200) were significantly enriched in the MF term for metal ion binding (*q* = 2.94e-02). The expression of these DEGs in WY was higher than in CS (**Figure 5C**) (**Supplementary Figure 7C**).

### 3.7 KOG functional classification of DEGs regulated by miRNA

After analyzing the targeting relationships between miRNAs and mRNAs, functional classification analysis was performed on the DEGs identified. The KOG functional classification of DE_miRNA-regulated DEGs was conducted. Focusing on the top five functional classes, four classes—T (signal transduction mechanisms), R (general function prediction only), O (posttranslational modification, protein turnover, chaperones), and J (translation, ribosomal structure, and biogenesis)—were shared by CS and WY in the comparison of 0 *vs*. 3-dpi. The fifth class differed, with class S (function unknown) identified in CS and class A (RNA processing and modification) identified in WY. However, in the 0 *vs*. 5-dpi and 3 *vs*. 5-dpi comparisons, the top five functional classes for both CS and WY were identical: T, R, O, G (carbohydrate transport and metabolism), and K (transcription) (**Supplementary Figure 8**). Similarly, the KOG functional classification of miRNA-regulated DEGs was performed. The results showed that the top five functional classes—T, R, O, G, and Q (secondary metabolites biosynthesis, transport, and catabolism)—were consistent across all three comparisons for both CS and WY (**Supplementary Figure 9**).

### 3.8 GO analysis of DEGs regulated by miRNA

GO analysis of DE_miRNA-regulated DEGs revealed 22, 18, and 17 functional classifications in the biological process (BP), cellular component (CC), and molecular function (MF) categories, respectively, for both CS and WY. Among these, the percentage of genes in 16 BP, 15 CC, and 12 MF classifications exceeded 0.1%. The results showed that most DEGs in the BP category were associated with metabolic processes, cellular processes, and single-organism processes. In the CC category, the majority of DEGs were located in the membrane, cell, cell part, membrane part, and organelle. In the MF category, most DEGs were related to catalytic activity and binding functions (**Supplementary Figure 10**). Similar findings were obtained from the GO analysis of miRNA-regulated DEGs (**Supplementary Figure 11**).

**Figure 6:**
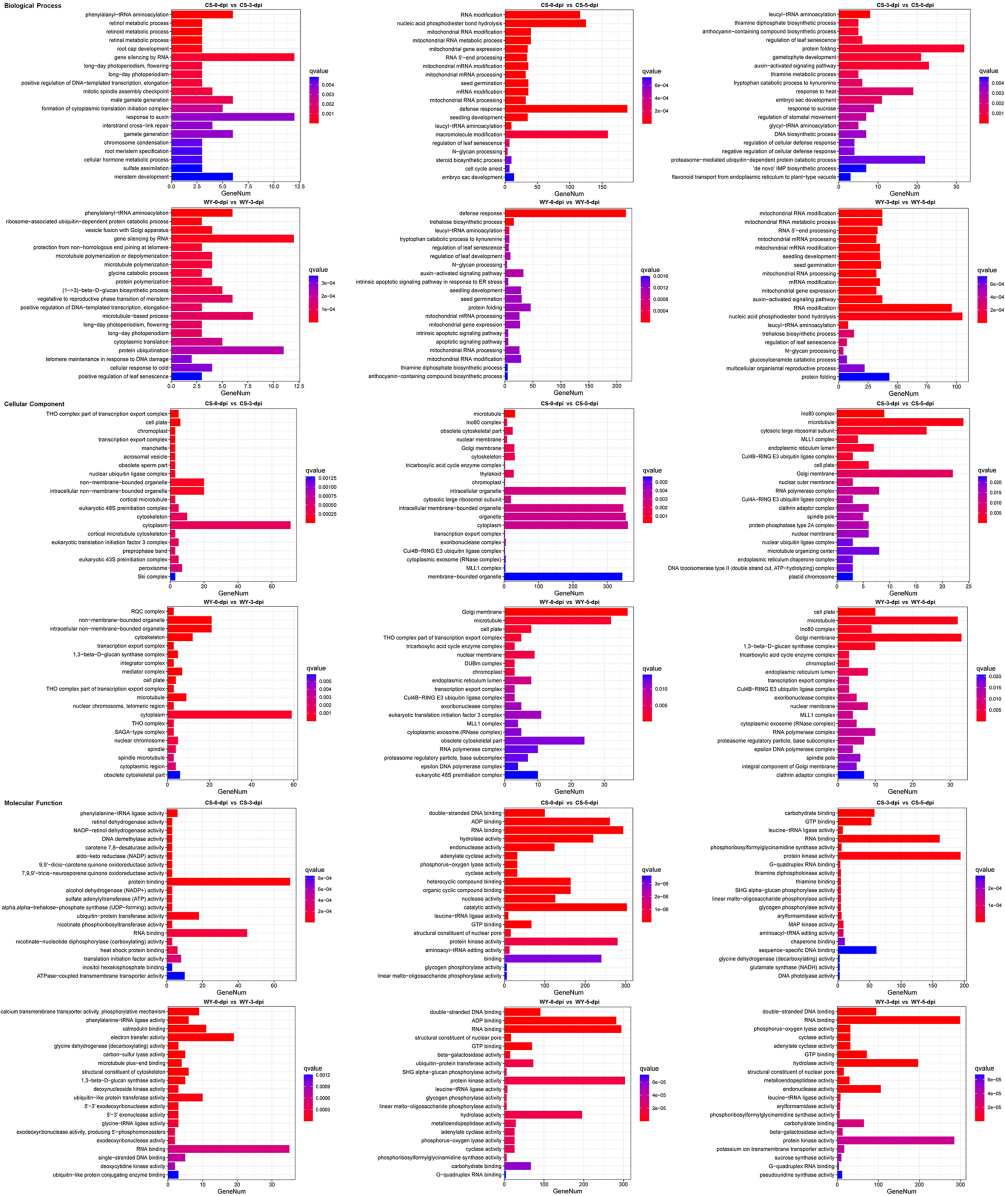
GO term enrichment analysis for DE_miRNA-regulated DEGs. GeneNum denotes the gene number.

GO enrichment analysis was conducted on the identified DEGs (**Dataset S7**, **Dataset S8**), with the top 20 terms for each comparison selected for display, using a *q*-value < 0.05 as the significance threshold. GO enrichment based on DE_miRNA-regulated DEGs revealed the most significant BP terms in CS were phenylalanyl-tRNA aminoacylation, RNA modification, and leucyl-tRNA aminoacylation for the 0 *vs*. 3-dpi, 0 *vs*. 5-dpi, and 3 *vs*. 5-dpi comparisons, respectively, whereas those in WY were phenylalanyl-tRNA aminoacylation, defense response, and mitochondrial RNA modification. The BP terms with the highest number of genes in CS included gene silencing by RNA, defense response, and protein folding, while those in WY were gene silencing by RNA, defense response, and nucleic acid phosphodiester bond hydrolysis. For the CC category, the most significant terms in CS were the THO complex (part of the transcription export complex), microtubule, and Ino80 complex, while those in WY were the ROC complex, Golgi membrane, and cell plate. The CC terms with the most genes in CS were cytoplasm (for the first two comparisons) and microtubule (for the third one), while in WY, the terms with the most genes were cytoplasm (for the first comparison) and Golgi membrane (for the subsequent two). In the MF category, the most significant terms in CS were phenylalanine-tRNA ligase activity, double-stranded DNA binding, and carbohydrate binding, while in WY they were calcium transmembrane transporter activity, phosphorylative mechanism, and double-stranded DNA binding. The terms with the most genes in CS were protein binding, catalytic activity, and protein kinase activity, whereas in WY, they were RNA binding, protein kinase activity, and RNA binding (**Figure 6**).

GO enrichment analysis based on miRNA-regulated DEGs revealed that the most significant BP terms in CS were protein-chromophore linkage, trehalose biosynthetic process, and leucyl-tRNA aminoacylation in the 0 *vs*. 3-dpi, 0 *vs*. 5-dpi, and 3 *vs*. 5-dpi comparisons, respectively. In WY, the most significant BP terms were protein-chromophore linkage in both 0 *vs*. 3-dpi and 0 *vs*. 5-dpi, and trehalose biosynthetic process for 3 *vs*. 5-dpi. The BP terms with the most genes in CS included defense response, carbohydrate metabolic process, and recognition of pollen, while in WY, they were defense response, photosynthesis, and response to oxidative stress. In the CC category, significant terms were observed only in two comparisons of WY (0 *vs*. 3-dpi and 0 *vs*. 5-dpi), with the most significant CC term being photosystem I in both cases. The CC terms with the most genes were the chloroplast thylakoid membrane for both comparisons, though this term was not statistically significant in the second comparison. In the MF category, the most significant MF term in the comparison of 0 *vs*. 3-dpi in CS was calmodulin binding, with the term having the most genes being protein kinase activity. In the other two comparisons (0 *vs*. 5-dpi and 3 *vs*. 5-dpi), the most significant MF term and the term with the most genes in CS were both protein kinase activity. For WY, the most significant MF terms in the 0 *vs*. 3-dpi and 0 *vs*. 5-dpi comparisons were chlorophyll binding and protein serine/threonine kinase activity, respectively. In both cases, the MF term with the most genes was protein kinase activity. In the 3 *vs*. 5-dpi comparison in WY, both the most significant MF term and the MF term with the most genes were protein serine/threonine kinase activity (**Supplementary Figure 12**).

### 3.9 KEGG analysis of DEGs regulated by miRNA

**Figure 7:**
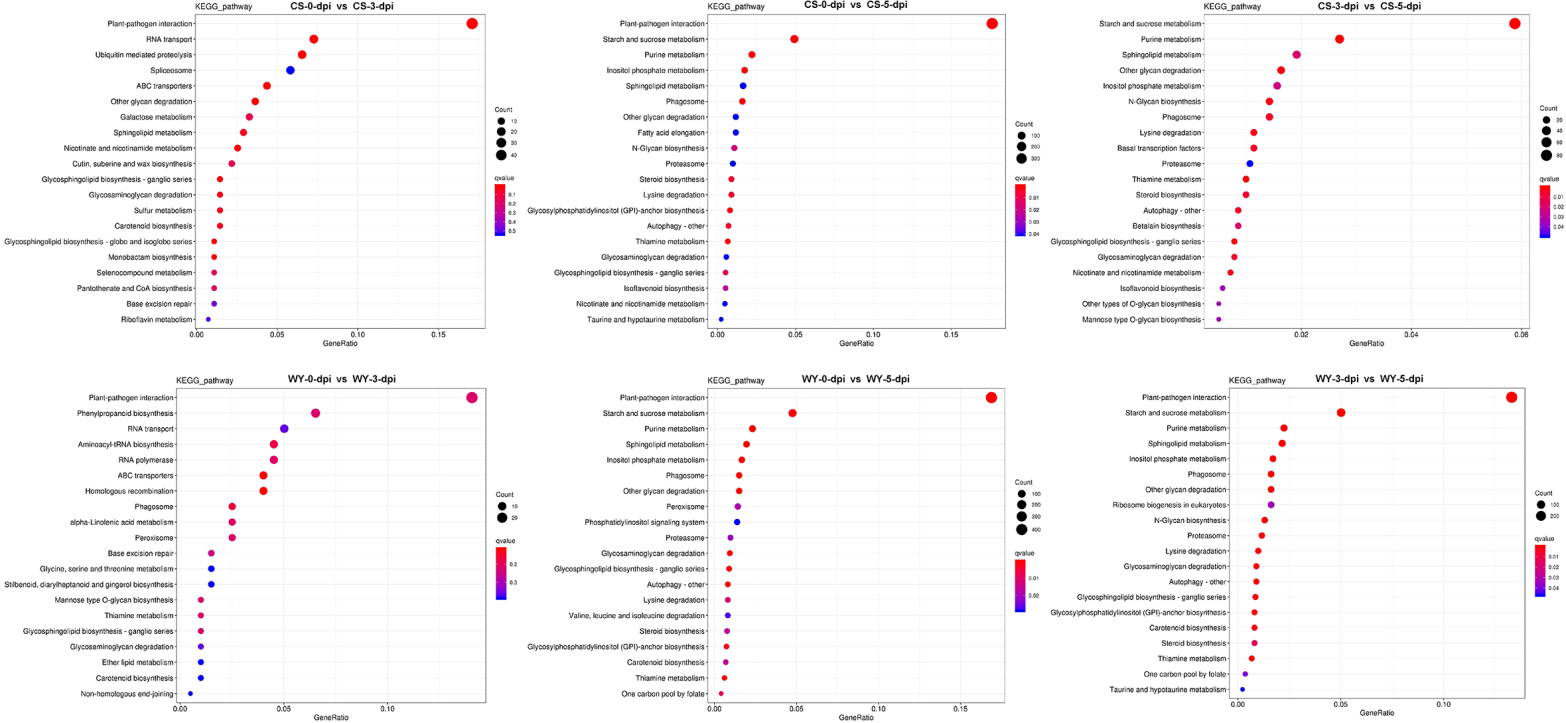
KEGG pathway enrichment analysis for DE_miRNA-regulated DEGs. GeneRatio indicates the gene ratio.

KEGG analysis based on DE_miRNA-regulated DEGs indicated that most metabolic pathways were within the metabolism category, with the pathway containing the most DEGs being the plant-pathogen interaction pathway (**Supplementary Figure 13**). Similar results were obtained from the KEGG analysis based on miRNA-regulated DEGs (**Supplementary Figure 14**). KEGG enrichment analysis was conducted on these DEGs (**Dataset S9**, **Dataset S10**), and the top 20 pathways for each comparison were selected for display, using *q*-value < 0.05 as the significance threshold. KEGG enrichment of DE_miRNA-regulated DEGs showed that the most significantly enriched pathway with the highest number of DEGs was the plant-pathogen interaction pathway across all comparisons, except for 3 *vs*. 5-dpi in CS, where the starch and sucrose metabolism pathway was the most significantly enriched (**Figure 7**). Similarly, KEGG enrichment based on miRNA-regulated DEGs also identified the plant-pathogen interaction pathway as the most significantly enriched pathway (**Supplementary Figure 15**).

To compare KEGG pathway enrichment between CS and WY at different time points, the top five pathways from each comparison were selected for further analysis. First, the top five KEGG pathways enriched based on DE_miRNA-regulated DEGs were examined. Results showed that only two pathways were identical between CS and WY in the 0 *vs*. 3-dpi comparison: plant-pathogen interaction (ko04626) and RNA transport (ko03013). However, these pathways were not significant in WY. The three specific pathways in CS were ubiquitin-mediated proteolysis (ko04120), spliceosome (ko03040; not significant), and ABC transporters (ko02010). In contrast, the three specific pathways in WY were phenylpropanoid biosynthesis (ko00940), aminoacyl-tRNA biosynthesis (ko00970), and RNA polymerase (ko03020), and none of them were significant. Additionally, both CS and WY had the same top five pathways for the 0 *vs*. 5-dpi comparison. For the 3 *vs*. 5-dpi comparison, four of the top five pathways were shared by CS and WY. The only differing pathway in CS was other glycan degradation (ko00511), while in WY, it was plant-pathogen interaction (ko04626) (**Table 2**).

**Table 2:** KEGG pathway enrichment analysis of DE_miRNA-regulated DEGs in CS and WY (top 5 pathways).

Comparison	Pathway ID	Description	*q*-value	Gene	DEG	Total DEG	DEG ratio
CS 0 *vs*. 3-dpi	ko04626	Plant-pathogen interaction	0.00375	6392	47	275	17.09%
	ko03013	RNA transport	0.00511	1979	20		7.27%
	ko04120	Ubiquitin mediated proteolysis	0.00511	1659	18		6.55%
	ko03040	Spliceosome	0.54038	2797	16		5.82%
	ko02010	ABC transporters	0.00375	776	12		4.36%
WY 0 *vs*. 3-dpi	ko04626	Plant-pathogen interaction	0.21824	6392	28	199	14.07%
	ko00940	Phenylpropanoid biosynthesis	0.21824	2419	13		6.53%
	ko03013	RNA transport	0.36091	1979	10		5.03%
	ko00970	Aminoacyl-tRNA biosynthesis	0.17576	1175	9		4.52%
	ko03020	RNA polymerase	0.20704	1267	9		4.52%
CS 0 *vs*. 5-dpi	ko04626	Plant-pathogen interaction	1.04e-25	6392	375	2127	17.63%
	ko00500	Starch and sucrose metabolism	2.21e-05	1910	105		4.94%
	ko00230	Purine metabolism	0.00073	754	47		2.21%
	ko00562	Inositol phosphate metabolism	0.00046	526	37		1.74%
	ko00600	Sphingolipid metabolism	0.04071	676	35		1.65%
WY 0 *vs*. 5-dpi	ko04626	Plant-pathogen interaction	1.17e-24	6392	415	2458	16.88%
	ko00500	Starch and sucrose metabolism	1.72e-05	1910	117		4.76%
	ko00230	Purine metabolism	2.64e-05	754	57		2.32%
	ko00600	Sphingolipid metabolism	0.00045	676	48		1.95%
	ko00562	Inositol phosphate metabolism	0.00022	526	41		1.67%
CS 3 *vs*. 5-dpi	ko00500	Starch and sucrose metabolism	7.38e-07	1910	83	1411	5.88%
	ko00230	Purine metabolism	0.00011	754	38		2.69%
	ko00600	Sphingolipid metabolism	0.01862	676	27		1.91%
	ko00511	Other glycan degradation	0.00232	440	20		1.63%
	ko00562	Inositol phosphate metabolism	0.02406	526	22		1.56%
WY 3 *vs*. 5-dpi	ko04626	Plant-pathogen interaction	7.72e-06	6392	297	2232	13.31%
	ko00500	Starch and sucrose metabolism	5.67e-06	1910	112		5.02%
	ko00230	Purine metabolism	0.00018	754	50		2.24%
	ko00600	Sphingolipid metabolism	5.44e-05	676	48		2.15%
	ko00562	Inositol phosphate metabolism	0.00024	526	38		1.70%

The KEGG pathways were enriched based on miRNA-regulated DEGs. In the 0 *vs*. 3-dpi comparison, four of the top five pathways were identical between CS and WY. However, the carbon metabolism pathway (ko02010) in CS and the starch and sucrose metabolism pathway (ko00500) in WY were not significant. The only differing pathway in CS was phenylpropanoid biosynthesis (ko00940), while in WY, it was photosynthesis (ko00195). For the other two comparisons (0 *vs*. 5-dpi, and 3 *vs*. 5-dpi), two of the top five pathways were different between CS and WY in each case, although the other three pathways—plant-pathogen interaction (ko04626), starch and sucrose metabolism (ko00500), and ABC transporters (ko02010)—were identical. In the 0 *vs*. 5-dpi comparison, the two different pathways in CS were flavonoid biosynthesis (ko00941) and glyoxylate and dicarboxylate metabolism (ko00630). In WY, the different pathways were carbon metabolism (ko01200) and photosynthesis (ko00195; not significant). In the 3 *vs*. 5-dpi comparison, the two different pathways in CS were flavonoid biosynthesis (ko00941) and cyanoamino acid metabolism (ko00460). In WY, the different pathways were phenylpropanoid biosynthesis (ko00940; not significant) and phenylalanine metabolism (ko00360) (**Table 3**).

**Table 3:** KEGG pathway enrichment analysis of miRNA-regulated DEGs in CS and WY (top 5 pathways).

Comparison	Pathway ID	Description	*q*-value	Gene	DEG	Total DEG	DEG ratio
CS 0 *vs*. 3-dpi	ko04626	Plant-pathogen interaction	8.29e-10	6392	108	550	19.64%
	ko00500	Starch and sucrose metabolism	9.02e-08	1910	45		8.18%
	ko00940	Phenylpropanoid biosynthesis	0.01981	2419	35		6.36%
	ko02010	Carbon metabolism	0.06503	1853	26		4.73%
	ko00941	Flavonoid biosynthesis	0.00015	763	18		3.27%
WY 0 *vs*. 3-dpi	ko04626	Plant-pathogen interaction	6.48e-10	6392	57	218	26.15%
	ko00941	Flavonoid biosynthesis	3.80e-07	763	16		7.34%
	ko00195	Photosynthesis	0.01866	1642	14		6.42%
	ko01200	Carbon metabolism	0.04673	1853	14		6.42%
	ko00500	Starch and sucrose metabolism	0.05285	1910	14		6.42%
CS 0 *vs*. 5-dpi	ko04626	Plant-pathogen interaction	1.49e-12	6392	270	1705	15.84%
	ko00500	Starch and sucrose metabolism	1.10e-15	1910	120		7.04%
	ko02010	ABC transporters	0.00101	776	40		2.35%
	ko00941	Flavonoid biosynthesis	0.00746	763	36		2.11%
	ko00630	Glyoxylate and dicarboxylate metabolism	0.00054	546	33		1.94%
WY 0 *vs*. 5-dpi	ko04626	Plant-pathogen interaction	3.65e-08	6392	150	900	16.67%
	ko00500	Starch and sucrose metabolism	3.65e-08	1910	63		7.00%
	ko01200	Carbon metabolism	0.00021	1853	50		5.56%
	ko00195	Photosynthesis	0.05300	1642	35		3.89%
	ko02010	ABC transporters	0.00015	776	28		3.11%
CS 3 *vs*. 5-dpi	ko04626	Plant-pathogen interaction	1.04e-11	6392	226	1378	16.40%
	ko00500	Starch and sucrose metabolism	2.56e-08	1910	85		6.17%
	ko02010	ABC transporters	7.54e-06	776	41		2.98%
	ko00941	Flavonoid biosynthesis	0.00166	763	33		2.39%
	ko00460	Cyanoamino acid metabolism	0.01322	700	28		2.03%
WY 3 *vs*. 5-dpi	ko04626	Plant-pathogen interaction	0.00223	6392	88	593	14.84%
	ko00500	Starch and sucrose metabolism	0.00032	1910	38		6.41%
	ko00940	Phenylpropanoid biosynthesis	0.07981	2419	34		5.73%
	ko00360	Phenylalanine metabolism	3.92e-05	635	21		3.54%
	ko02010	ABC transporters	0.00034	776	21		3.54%

### 3.10 RT−qPCR validation of DEGs regulated by miRNA

**Figure 8:**
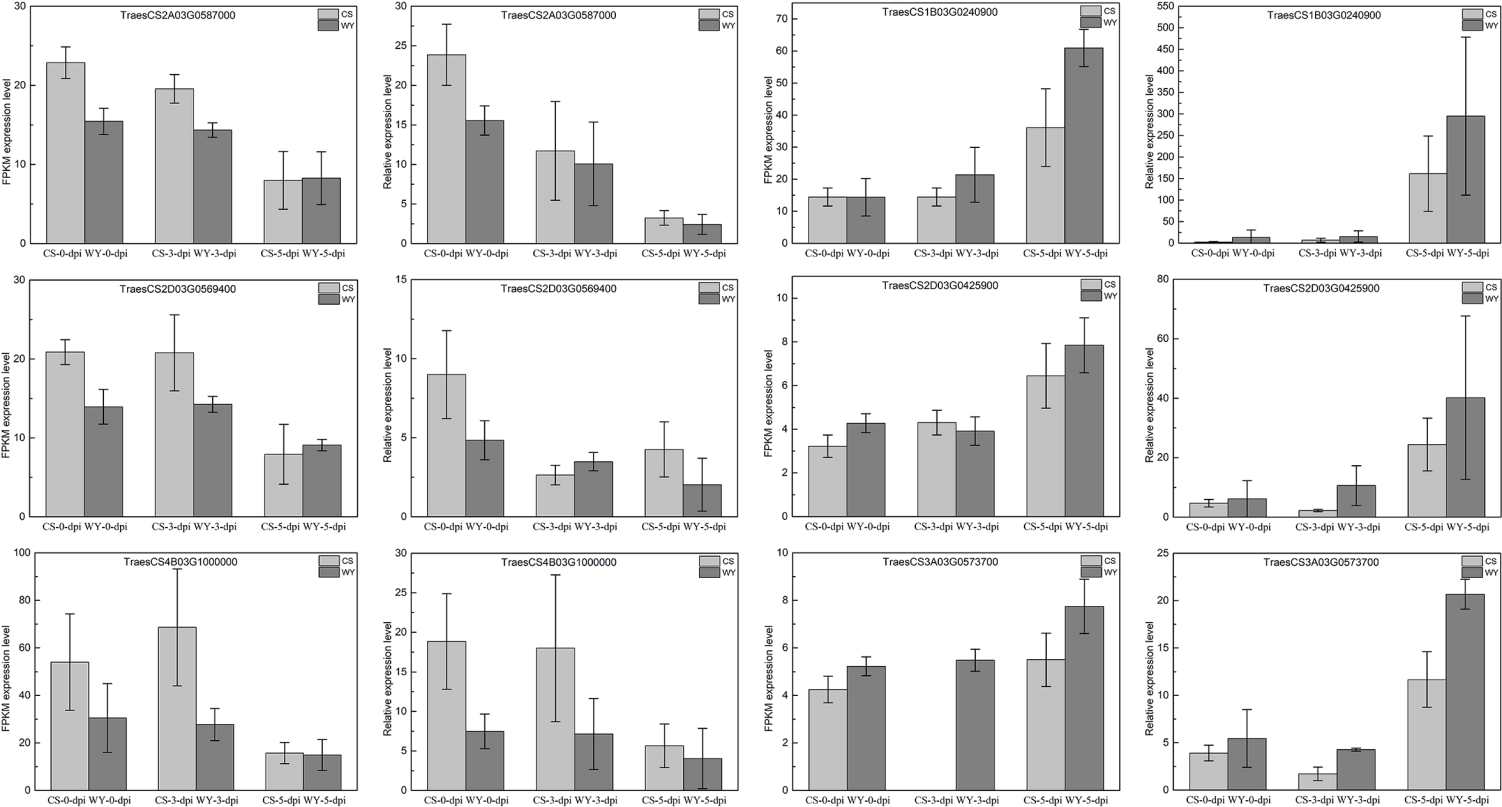
Relative expression comparison for selected genes using RNA-seq and RT-qPCR data.

Based on the integrated analysis of the targeting relationships between miRNA and mRNA, two DE_miRNAs—tae-miR1122a and novel_miR_228—were identified from the top 20 DE_miRNAs and were specifically expressed in WY and CS at 5-dpi. Six DEGs, three with a negative regulatory relationship with tae-miR1122a and the other three with novel_miR_228, were selected for RT-qPCR validation of their expression levels. The DEGs targeted by tae-miR1122a were TraesCS1B03G0240900, TraesCS2D03G0425900, and TraesCS3A03G0573700, associated with the KEGG pathway of glutathione metabolism (ko00480), phagosome pathway (ko04145), and autophagy-other (ko04136), respectively. The DEGs targeted by novel_miR_228 were TraesCS2A03G0587000, TraesCS2D03G0569400, and TraesCS4B03G1000000, and both of the first two were associated with the plant-pathogen interaction pathway (ko04626), and the third one had no KEGG pathway annotation. The relative expression data from RT-qPCR for the selected genes were compared with the RNA-seq analysis data, and the results revealed that the expression patterns were largely consistent between the two methods, confirming that the RNA-seq analysis in this study was reliable and suitable for further research (**Figure 8**).

## 4 Discussion

### 4.1 Transcriptomic study for FHB resistance

Fusarium head blight (FHB) significantly threatens wheat production globally, leading to yield loss and grain contamination by mycotoxins. Breeding and utilizing FHB-resistant wheat cultivars provide effective and eco-friendly solutions to mitigate these effects. A comprehensive understanding of the molecular mechanisms underlying wheat-pathogen interactions has laid the genetic foundation for wheat breeding programs aimed at combating FHB. Recent transcriptomic studies advanced our knowledge of key molecular pathways associated with FHB resistance and susceptibility (Kazan and Gardiner, 2018), and will aid in identifying key genes and mechanisms involved in the plant’s defense response. For example, previous studies have shown that hormone biosynthesis and signal transduction pathways actively respond to FHB infection, and contribute to plant defense reaction (Biselli et al., 2018). Specifically, salicylic acid (SA) and jasmonic acid (JA) positively affect FHB resistance, while auxin and abscisic acid (ABA) are linked to susceptibility of FHB. Ethylene has dual roles in the interaction with *F. graminearum* (Wang et al., 2018). Additionally, resistant genotypes exhibit early and intense expression of defense-related genes, including those involved in redox homeostasis and secondary metabolite biosynthesis (Xiao et al., 2013). Differentially expressed miRNAs and lncRNAs play roles in regulating gene expression related to biotic and abiotic stress responses, respectively (Biselli et al., 2018; Soresi et al., 2023). Here, a comprehensive transcriptomic analysis of two Sichuan wheat landraces with contrasting FHB resistance (WY was resistant, while CS was susceptible) was conducted based on the prior research (Wan et al., 1997). Analysis of the expression and regulatory networks of lncRNAs, circRNAs, mRNAs, and miRNAs provides a detailed view of the regulatory differences between resistant and susceptible genotypes.

### 4.2 Differential expression of lncRNAs, circRNAs, and mRNAs

Our findings reveal significant differences in RNA expression profiles between WY and CS at different stages post-inoculation (**Figures 1**, **2**). The differential expression of lncRNAs is particularly noteworthy. Previous studies have indicated that lncRNAs modulate the expression of defense-related genes, such as those involved in the JA pathway, which is crucial for plant disease resistance (Chen et al., 2023). Specific lncRNAs, like ALEX1 in rice, enhance resistance to bacterial blight by activating JA signaling and increasing JA content (Yu et al., 2019). Furthermore, lncRNAs can function as ceRNAs, decoying miRNAs to regulate the expression of target genes involved in immune responses. For instance, lncRNAs in tomato modulate MYB transcription factors by decoying miR159, enhancing resistance to *Phytophthora infestans* (Cui et al., 2020). LncRNAs have also been shown to regulate pathogen resistance and often peak early during pathogen invasion (Duan et al., 2020). Our results confirm this, with WY and CS displaying substantial lncRNA activity at 3 and 5-dpi, suggesting that early lncRNA activity is crucial for an effective defense against *F. graminearum*. Moreover, the greater number of differentially expressed lncRNAs observed in the field conditions compared to greenhouse settings suggests that environmental factors significantly influence lncRNA-mediated responses (Soresi et al., 2023).

In addition to lncRNAs, which are known for their regulatory roles, the circRNAs have emerged as another crucial class of regulatory RNAs. The role of circRNAs in responding to FHB is still not well-defined. Our data showed limited differential expression of circRNAs in both genotypes (**Figures 1**, **2**), aligning with findings that indicate a reduction in circRNA expression following *F. graminearum* infection, possibly due to their involvement in fine-tuning gene expression during initial infection stages (Yin et al., 2022). Together, these results suggest that lncRNAs actively mediate gene regulation during early infection, while circRNAs may play a more subdued role, potentially involved in more sustained immune responses.

The differentially expressed mRNAs was more pronounced in CS than in WY (**Table 1**), consistent with the previous studies that susceptible genotypes often exhibit a broader transcriptional response. This reflects a less-targeted response, activating a wide array of genes rather than focusing on specific defense-related pathways (Erayman et al., 2015). In contrast, resistant genotypes like WY likely mount a more efficient and focused response, resulting in fewer DEGs but potentially more effective pathogen resistance mechanisms (Walker et al., 2024).

### 4.3 Integrated analysis of targeting relationships among RNAs

To further understand the regulatory roles of these RNAs, we analyzed their interactions and targeting relationships. Focusing on miRNAs as central regulators, we observed primary targeting relationships with mRNAs (**Figure 3**), particularly at 3 and 5 dpi. This miRNA-mRNA interaction network suggests that miRNAs play a vital role in silencing pathogen-related genes, curbing the spread of infection and enhancing plant resistance (Jiao and Peng, 2018; MaChado et al., 2018; Fu et al., 2023). In contrast, fewer interactions were observed when lncRNAs or circRNAs were analyzed as the central regulators (**Supplementary Figures 2**, **Supplementary Figures 3**). This lack of significant ceRNA relationships at later infection stages indicates that while lncRNAs and circRNAs may be important during initial pathogen recognition, their influence diminishes as the infection progresses. This temporal division of labor highlights miRNAs as the primary regulatory players in sustained immune responses, while lncRNAs and circRNAs contribute more to the early stages of infection.

### 4.4 Differential expression of up-regulated genes and their role in wheat FHB resistance

The differential expression analysis showed that CS had more up-regulated DEGs than WY across all comparisons (**Figures 4A, B**). In CS, the number of up-regulated genes exceeded the down-regulated ones at all time points, indicating a stronger overall transcriptional response to infection. However, this response was likely non-specific and may contribute to the higher susceptibility of CS to FHB. In contrast, WY displayed more balanced gene expression, with up-regulated DEGs outnumbering down-regulated ones only at 3 *vs*. 5 dpi. This suggests that while both genotypes mount a defense response to *F. graminearum*, a broader transcriptional activation of CS may lead to an inflammatory response that fails to efficiently control pathogen spread (Pan et al., 2018; Erayman et al., 2015).

The GO term enrichment analysis revealed differences in the molecular functions (MF) of the up-regulated DEGs between CS and WY (**Figure 4C**). At 0 *vs*. 3 dpi, CS showed enrichment for phenylalanine ammonia-lyase activity and heme binding, both of which are important for phenylpropanoid biosynthesis and managing oxidative stress. These pathways help protect the plant from damage and activate secondary metabolites involved in defense against fungal pathogens (Ramaroson et al., 2022). In contrast, WY was enriched for hydrolase activity and polysaccharide binding, suggesting a more localized, mechanical defense, likely related to altering the cell wall to prevent fungal invasion. At 0 *vs*. 5 dpi, CS enriched for glutathione transferase activity and iron ion binding, both involved in detoxifying reactive oxygen species (ROS) and maintaining redox balance. These processes are crucial during pathogen infection (Van Breusegem et al., 2008). WY, on the other hand, showed enrichment for ATPase-coupled transmembrane transporter activity and ATPase activity, which are involved in ion transport and energy metabolism, possibly indicating a different or less efficient stress response. At 3 *vs*. 5 dpi, CS showed enrichment for protein kinase activity and protein serine/threonine kinase activity, both crucial for signalling immune responses (Pan et al., 2018; Jiang et al., 2022). The presence of phenylalanine ammonia-lyase activity in CS also suggests ongoing activation of pathways important for producing lignin and antimicrobial compounds. WY, by contrast, was enriched for glutathione transferase activity, heme binding, and UDP-glycosyltransferase activity, which are involved in detoxification, ROS management, and modifying secondary metabolites (Wang et al., 2020a; Gharabli et al., 2023; Walker et al., 2024). These differences highlight the more specialized defense mechanisms in WY, which may explain its greater resistance to FHB.

The KEGG pathway enrichment analysis highlighted further differences in the defense mechanisms of CS and WY (**Figure 4D**). At 0 *vs*. 3 dpi, CS showed enrichment in the biosynthesis of amino acids pathway, which is important for producing amino acids needed for protein synthesis and secondary metabolite formation. In contrast, WY enriched for plant hormone signal transduction, suggesting that hormonal regulation (such as JA and SA) plays a key role in its early defense response (Pieterse et al., 2012; Pan et al., 2018). This supports the idea that the resistance of WY to FHB may involve a more coordinated immune response, with specific hormones activated by fungal infection. At 0 *vs*. 5 dpi, CS enriched for pathways related to phenylpropanoid biosynthesis and carbon metabolism, both crucial for stress responses, secondary metabolite production, and energy balance during infection. In contrast, WY enriched for glutathione metabolism and ABC transporters, which help detoxify oxidative stress and remove harmful substances (Wang et al., 2020b; Gullner et al., 2018). This suggests that the resistance of WY may depend more on detoxification and transporting stress-related compounds. At 3 *vs*. 5 dpi, the differences were even more pronounced. CS enriched for plant-pathogen interaction and amino acid biosynthesis, which are important for immune recognition and defense protein synthesis. WY, however, enriched for glutathione metabolism and ABC transporters, helping maintain cellular integrity during stress (Wang et al., 2020b; Gullner et al., 2018). Additionally, WY showed enrichment in flavonoid biosynthesis and phenylalanine metabolism, which suggests a shift towards producing secondary metabolites as a defense strategy to limit pathogen growth.

A detailed analysis of the up-regulated genes in the glutathione metabolism pathway during the 3 *vs*. 5 dpi comparison in WY identified 93 DEGs, of which 80 were shared between CS and WY (**Figure 4E**). These genes are involved in detoxifying ROS and maintaining cellular redox balance, which are essential for plant defense against oxidative stress caused by fungal infection. Interestingly, 13 DEGs were only differentially expressed in WY, suggesting a unique resistance mechanism in this genotype. The heatmap analysis showed that two genes, TraesCS2B03G1525600 and TraesCS6B03G0425000, had significantly higher expression levels in CS compared to WY, which may indicate a larger-scale response in CS, but this doesn’t necessarily mean a more effective defense. The differences in gene expression highlight the distinct defense strategies between the two genotypes, with WY showing a more localized, focused response to oxidative stress.

### 4.5 Integrated analysis of miRNA-mRNA targeting relationships

The results show a clear difference in miRNA profiles between the two wheat genotypes, especially at different time points after infection. At 0 *vs*. 3 dpi, only two miRNAs were common between CS and WY, indicating that each genotype activates different miRNA pathways early in the infection. However, by 0 *vs*. 5 dpi and 3 *vs*. 5 dpi, 16 and 15 miRNAs were shared between the genotypes, suggesting a more similar response as the infection progresses. Notably, novel_miR_228 and tae-miR1122a were key miRNAs with different roles in the two genotypes. Novel_miR_228, which was up-regulated in CS, down-regulated 28 and 23 genes related to defense in CS at 0 *vs*. 5 dpi and 3 *vs*. 5 dpi, respectively (**Figure 5B**) (**Supplementary Figure 7B**). This suggests that it may weaken the defense response of host, making CS more susceptible to FHB. On the other hand, tae-miR1122a, which was down-regulated in WY, up-regulated 27 and 23 defense-related genes in WY at the same time points, suggesting that it helps enhance resistance in WY. These findings support the idea that miRNAs play a role in regulating the defense mechanisms of host in response to fungal stress, contributing to FHB resistance (MaChado et al., 2018; Jin et al., 2020).

The GO analysis of the DEGs regulated by the identified miRNAs revealed important molecular functions linked to FHB resistance. In CS, novel_miR_228 down-regulated genes related to key functions like histone acetyltransferase activity and DNA binding, which are essential for transcription and DNA repair. This down-regulation could weaken the ability of host to manage gene expression and repair DNA, making it more vulnerable to fungal damage. Genes like TraesCS1A03G0367900 and TraesCS6B03G0592100 showed strong associations with these functions, highlighting how *F. graminearum* might exploit these weaknesses to infect the host. In contrast, in WY, tae-miR1122a up-regulated genes involved in metal ion binding, such as TraesCS2B03G0537600, TraesCS2D03G0425900, and TraesCS6A03G0855700 (**Figure 5C**) (**Supplementary Figure 7C**). These genes help maintain cellular stability under stress, suggesting that they play a role in the defense of plants (Wang et al., 2020b). The up-regulation of these genes in WY may support antioxidant and detoxification pathways, helping the plant cope with the oxidative damage caused by fungal infection (Gullner et al., 2018). The significant enrichment of these genes in WY points to their potential role in strengthening the ability of host to resist fungal stress by binding toxic ions released by the pathogen, thus helping maintain cellular integrity and defense against FHB.

The comparison between WY and CS shows a clear difference in how they respond to *F. graminearum* infection. In WY, the expression of defense-related genes is stronger, with down-regulation of miRNAs like tae-miR1122a and up-regulation of their target genes involved in metal ion binding and stress responses. This suggests that the resistance of WY to FHB may be due to better regulation of metal ion balance, oxidative stress, and immune signaling, all of which are essential for protecting cells during infection. In CS, the up-regulation of novel_miR_228 and the down-regulation of key defense genes indicate a weaker response to the fungal infection. This may explain why CS, a more susceptible genotype, struggles to mount a strong defense against *F. graminearum*, making it more prone to disease.

### 4.6 GO term and KEGG pathway enrichment of DEGs regulated by miRNA

GO and KEGG pathway analyses further illustrate the functional divergence between WY and CS in response to FHB. In GO classification, the DEGs of WY were enriched in defense-related categories, including gene silencing by RNA and defense response (**Figure 6**). This enrichment suggests that WY is genetically predisposed to actively respond to pathogen invasion, prioritizing immune functions over general metabolic processes. In the cellular component (CC) category, the DEGs of WY were primarily localized to membrane and cytoplasmic components, critical sites for pathogen recognition and signaling. In contrast, CS displayed a more generalized distribution across organelles, indicative of a less-targeted defense approach.

KEGG pathway enrichment revealed that immune pathways were central to the response of WY, particularly the plant-pathogen interaction pathway (**Figure 7**). This pathway was consistently enriched across all time points, reflecting a robust immune response in WY, channeling resources toward defense mechanisms upon pathogen detection (Zaynab et al., 2018). Conversely, the response of CS emphasized starch and sucrose metabolism pathways (**Table 2**), suggesting a baseline metabolic stability rather than a targeted immune response. This finding is consistent with previous studies that susceptible genotypes often allocate resources to general metabolism at the expense of pathogen-specific defenses (Brauer et al., 2019).

Additionally, pathways like phenylpropanoid biosynthesis and phenylalanine metabolism were enriched in WY but not in CS (**Table 3**). These pathways are known to produce secondary metabolites that strengthen structural defenses and stress responses, underscoring the preparedness of WY for pathogen attack (Zaynab et al., 2018). The divergence in pathway enrichment between WY and CS highlights how WY allocates resources more strategically, channeling efforts toward immune functions while CS appears to prioritize metabolic maintenance. This strategic resource allocation likely contributes to enhancing the resistance of WY to FHB.

### 4.7 Role of miRNAs in regulating defense mechanisms

This study highlighted the critical role of miRNAs in response to FHB resistance. miRNAs are known to target specific mRNAs, modulating gene expression in response to stressors, including pathogens. For example, miRNAs are integral to the defense mechanisms of plants against pathogens. They regulate the expression of genes involved in phytohormone signaling, ROS production, and nucleotide-binding site-leucine-rich repeat (NBS-LRR) gene expression, which are critical for mounting an effective defense response (Yang et al., 2021). The interaction between miRNAs and lncRNAs complicated this regulatory network, further fine-tuning the immune response of plants (Song et al., 2021). We found that WY exhibited a notably stronger miRNA response than CS, particularly at later infection stages (**Table 1**). miRNAs in WY were enriched in defense-related pathways such as glutathione metabolism and plant-pathogen interactions, crucial for oxidative stress regulation—an essential factor in plant defense that limits pathogen spread (Dorion et al., 2021). The enrichment of miRNAs in plant-pathogen interaction pathways supports their role in enhancing immune responses, allowing WY to recognize and respond more effectively to *F. graminearum* invasion. In contrast, miRNA pathways in CS were predominantly linked to general metabolic processes rather than defense-specific responses, suggesting that the miRNAs of CS may not be primed for targeted pathogen defense. This aligns with previous studies that susceptible genotypes often prioritize maintaining metabolic homeostasis over rapid immune activation, leaving them more vulnerable to pathogens (Brauer et al., 2019). These differences indicate that miRNAs in resistant genotypes like WY are more adept at regulating specific pathways linked to pathogen defense, facilitating a faster and more efficient immune response.

In summary, this study provides valuable insight into the molecular mechanisms underlying FHB resistance in two Sichuan wheat landraces WY (HR) and CS (HS). Through a comprehensive transcriptomic analysis, we identified distinct differences in how these two genotypes respond to *F. graminearum*. The resistant genotype, WY, showed a targeted miRNA response, particularly at later infection stages, which effectively regulated pathways important for defense, such as glutathione metabolism and phenylpropanoid biosynthesis. In contrast, the susceptible genotype, CS, exhibited a broader transcriptional response focused more on general metabolism than specific pathogen defense. Our findings emphasize the critical role of miRNA-mRNA interactions in enhancing the resistance of WY. Although lncRNAs and circRNAs were also involved in resistance, their contributions were limited in comparison with miRNAs. This research not only emphasized the importance of miRNA-mediated defense mechanisms but also suggested potential molecular targets to improve FHB resistance in breeding programs. Future studies should focus on validating these pathways and incorporating deep learning method and genomic selection (GS) (Chen et al., 2024; Wang et al., 2024b; Ma et al., 2024) techniques to develop more resilient wheat varieties, ensuring sustainable wheat production amid ongoing challenges posed by FHB disease.

## Data Availability

The original contributions presented in the study are publicly available. This data can be found here: NCBI, PRJNA1183537.
