# Supplementary material for: Transcriptomic analysis of two Chinese wheat landraces with contrasting Fusarium head blight resistance reveals miRNA-mediated defense mechanisms
Source: Front Plant Sci. 2025 Feb 28;16:1537605. doi: 10.3389/fpls.2025.1537605 (PMC11906714; doi:10.3389/fpls.2025.1537605)
Supplement: Supplementary file 11 [file DataSheet11.docx]

Supplementary Material

## Supplementary Figures


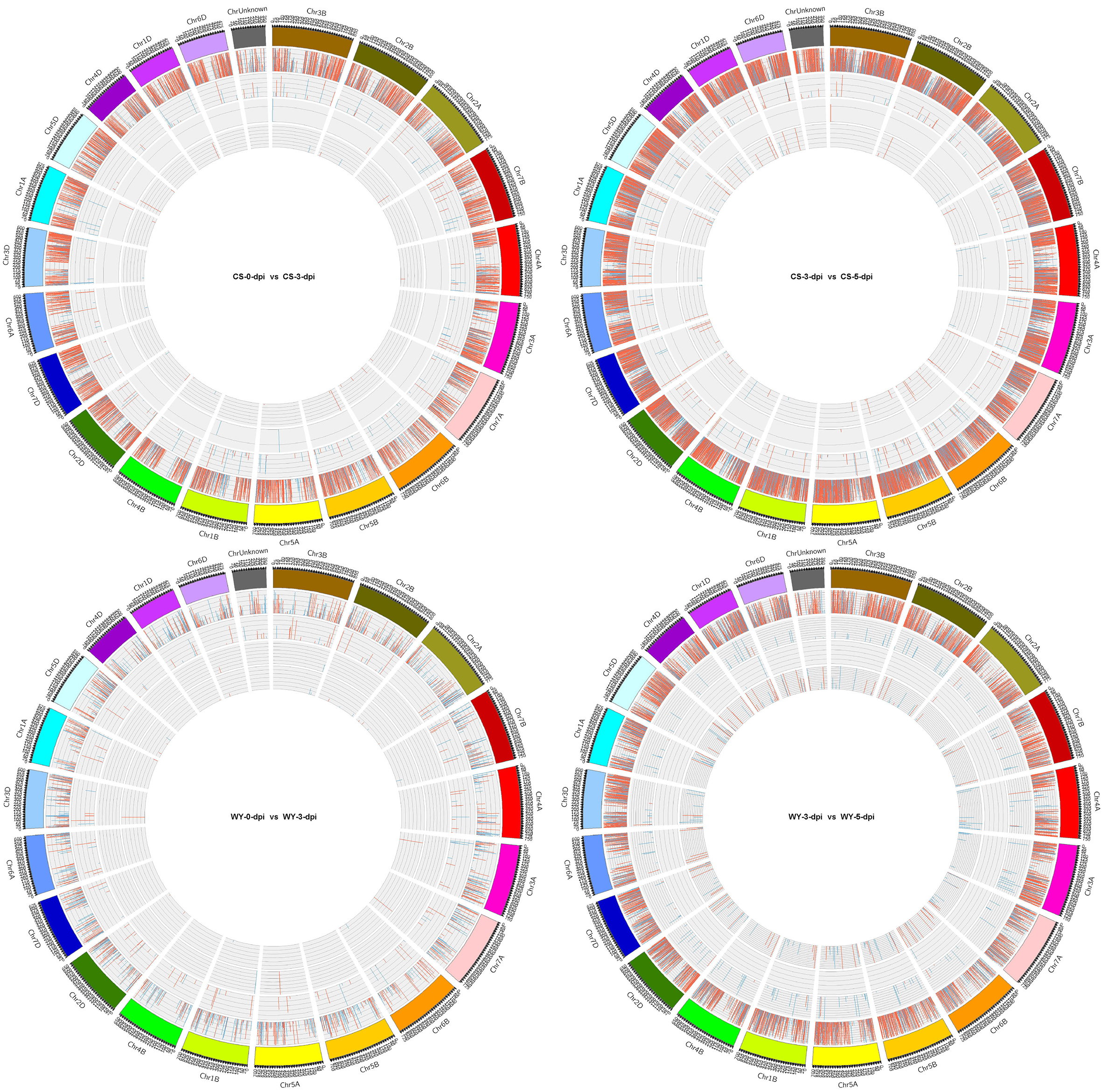


**Supplementary Figure 1.** Expression profiles of differentially expressed RNAs across various comparisons. The outermost ring shows chromosome information, followed by mRNA (gene), lncRNA, circRNA, and miRNA. For each group of differentially expressed RNAs, red, blue, and the height indicate up-regulation, down-regulation, and significance (-log_10_ (FDR)), respectively.


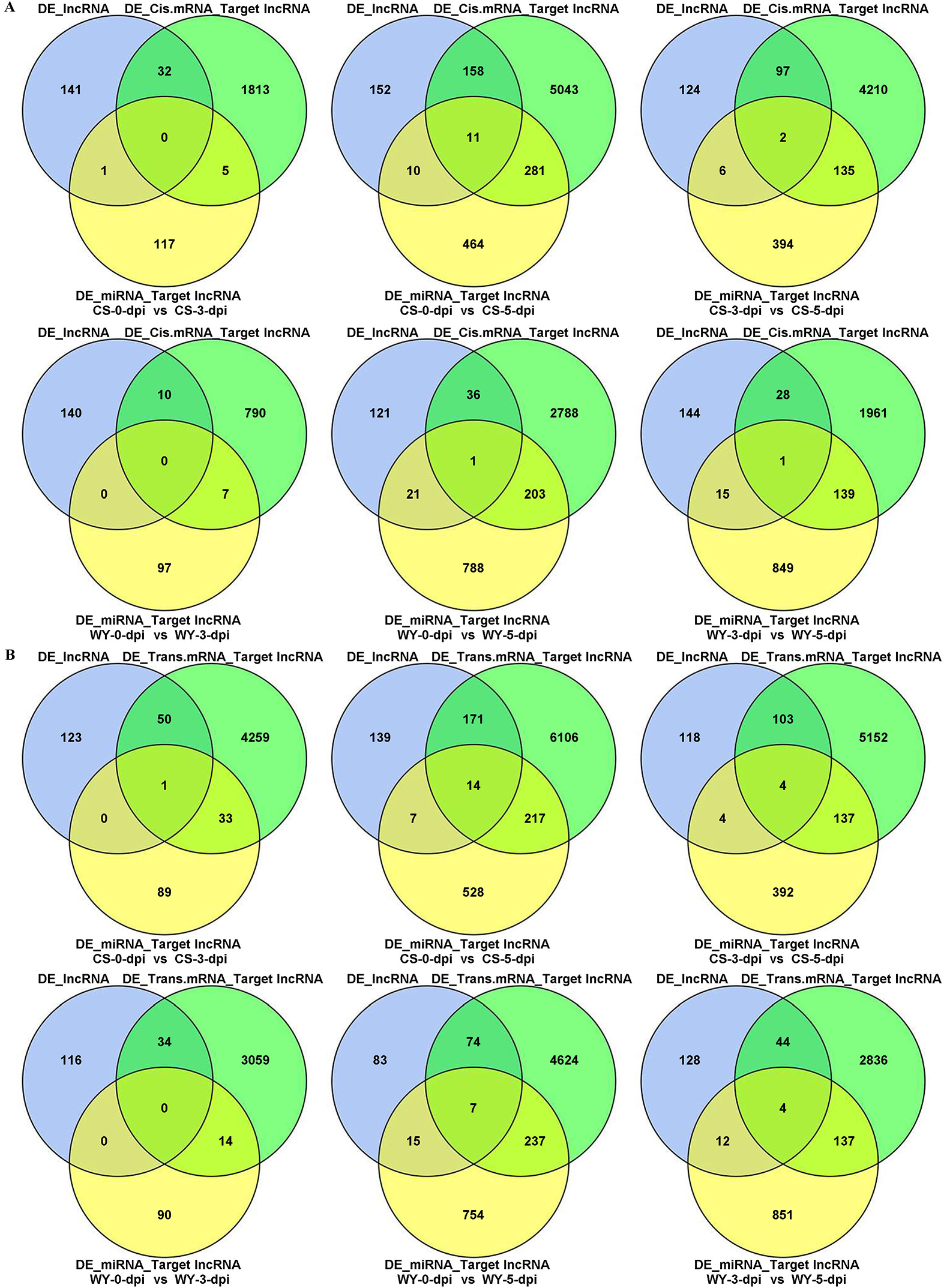


**Supplementary Figure 2.** Interaction of differentially expressed lncRNAs with all lncRNAs targeted by differentially expressed mRNAs and miRNAs. Here, DE_lncRNA represents differentially expressed lncRNAs; (**A**) DE_Cis.mRNA_Target lncRNA and (**B**) DE_Trans.mRNA_Target lncRNA represent cis- and trans-targeted lncRNAs by differentially expressed mRNAs; and DE_miRNA_Target lncRNA represents lncRNAs targeted by differentially expressed miRNAs.

**
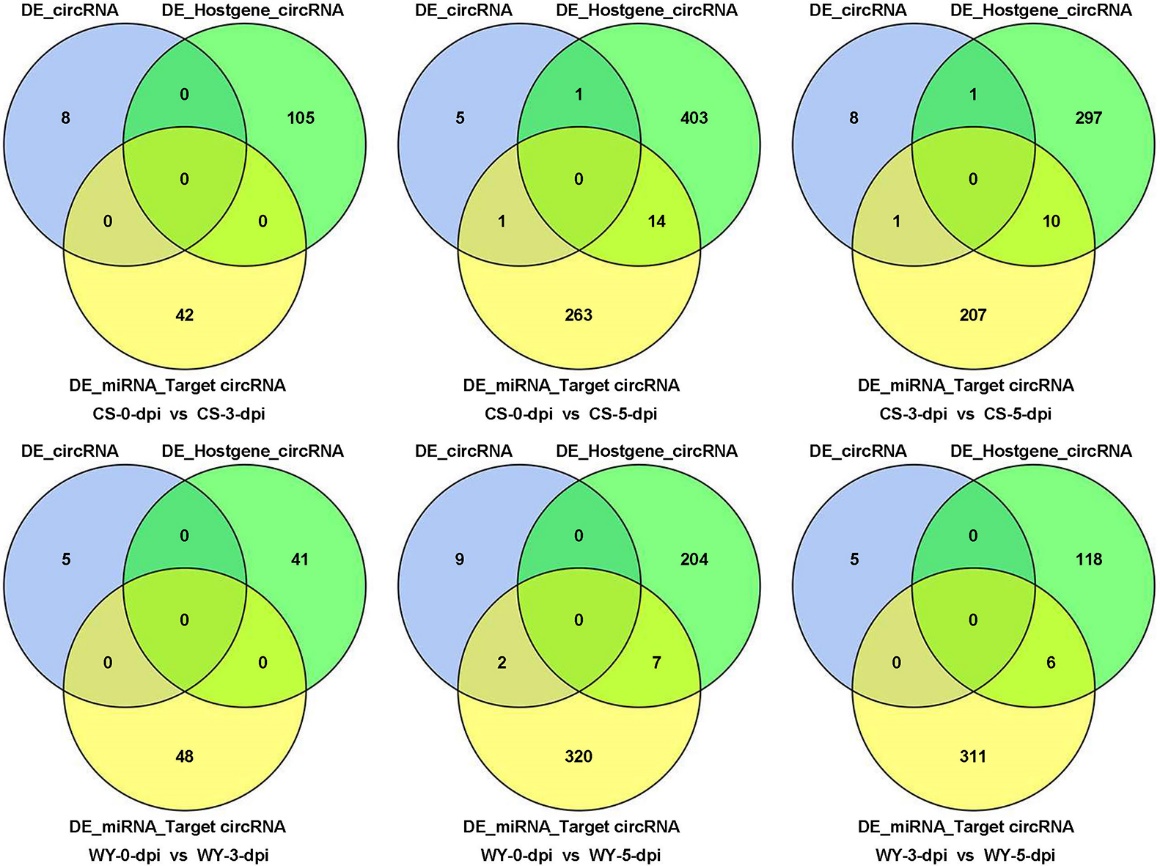
**

**Supplementary Figure 3.** Interaction of differentially expressed circRNAs (DE_circRNA) with circRNAs linked to differentially expressed genes as host genes (DE_Hostgene_circRNA) and circRNAs targeted by differentially expressed miRNAs (DE_miRNA_Target circRNA).


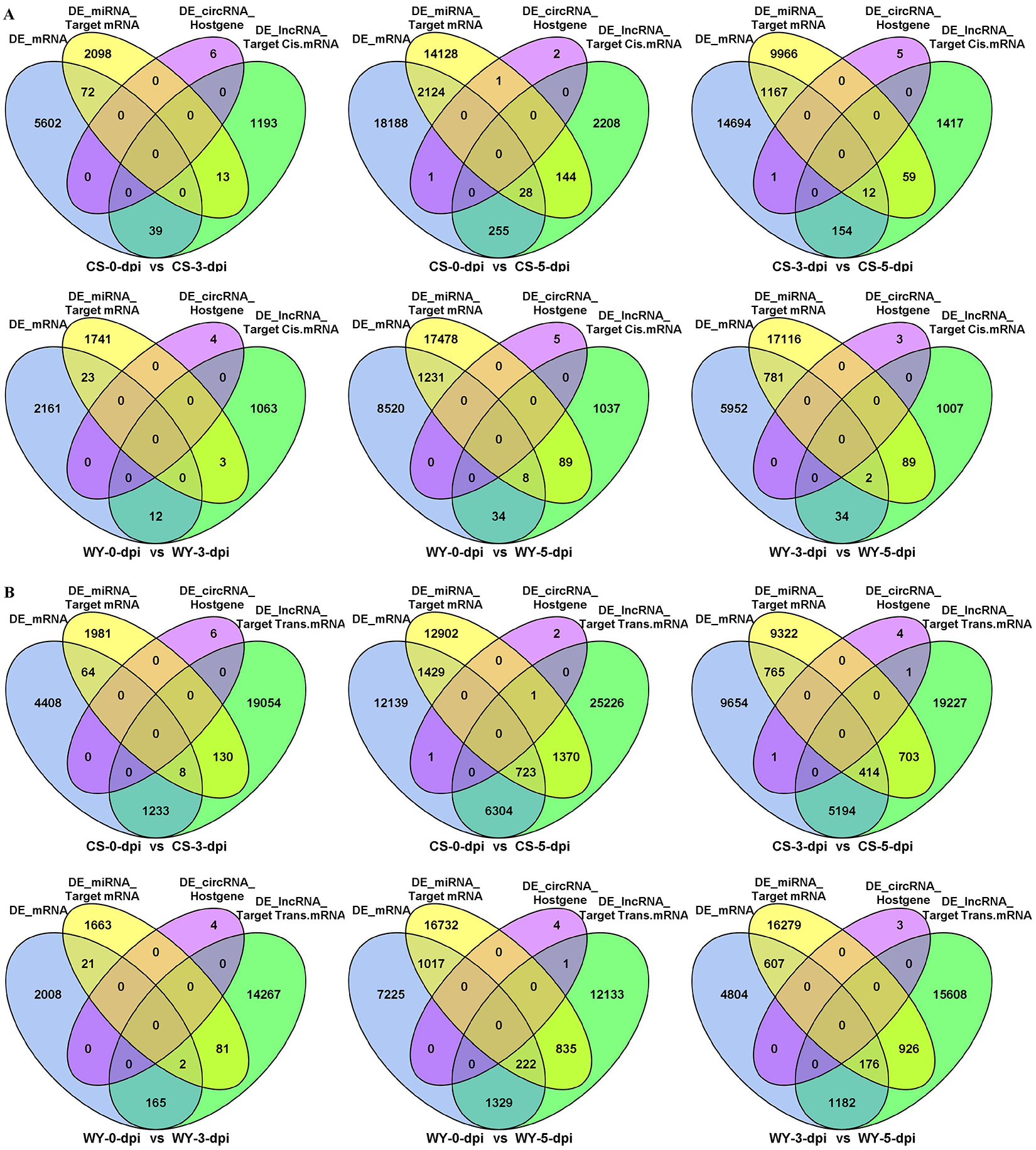


**Supplementary Figure 4.** Interaction of differentially expressed mRNAs (DEGs) with all mRNAs targeted by differentially expressed miRNAs, lncRNAs, and circRNAs. Here, DE_mRNA represents the differentially expressed genes; DE_miRNA_Target mRNA represents genes targeted by differentially expressed miRNAs; and (**A**) DE_lncRNA_Target Cis.mRNA and **(B)** DE_lncRNA_Target Trans.mRNA represent genes targeted by differentially expressed cis- or trans-acting lncRNAs, respectively.

**
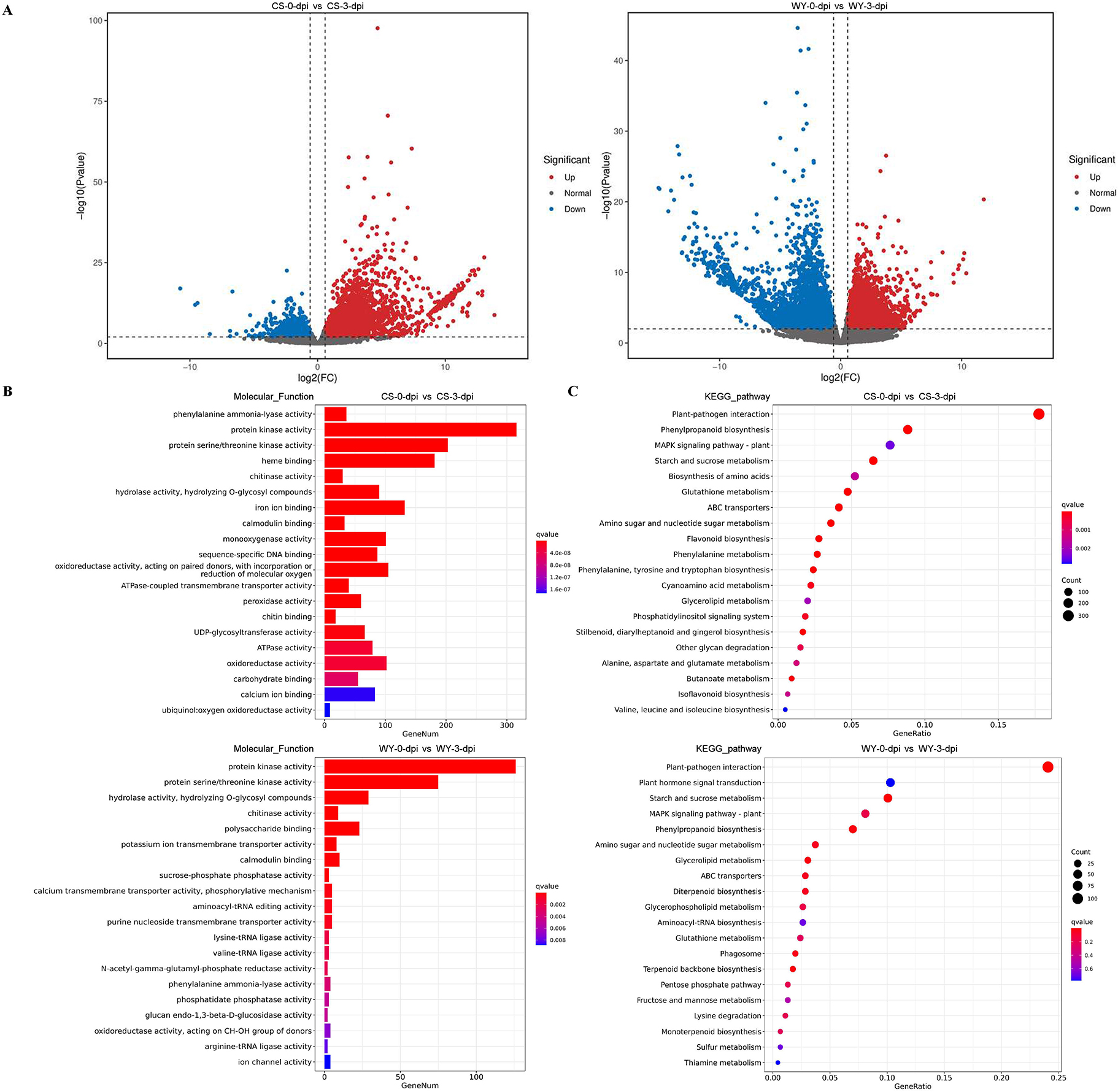
**

**Supplementary Figure 5.** Differential expression analysis for up-regulated genes collected at 3-dpi with *F. graminearum*. (**A**) Volcano plot of DEGs obtained in the comparison of 0 vs. 3-dpi. (**B**) GO term and (**C**) KEGG pathway enrichment analysis for significantly up-regulated DEGs.

**
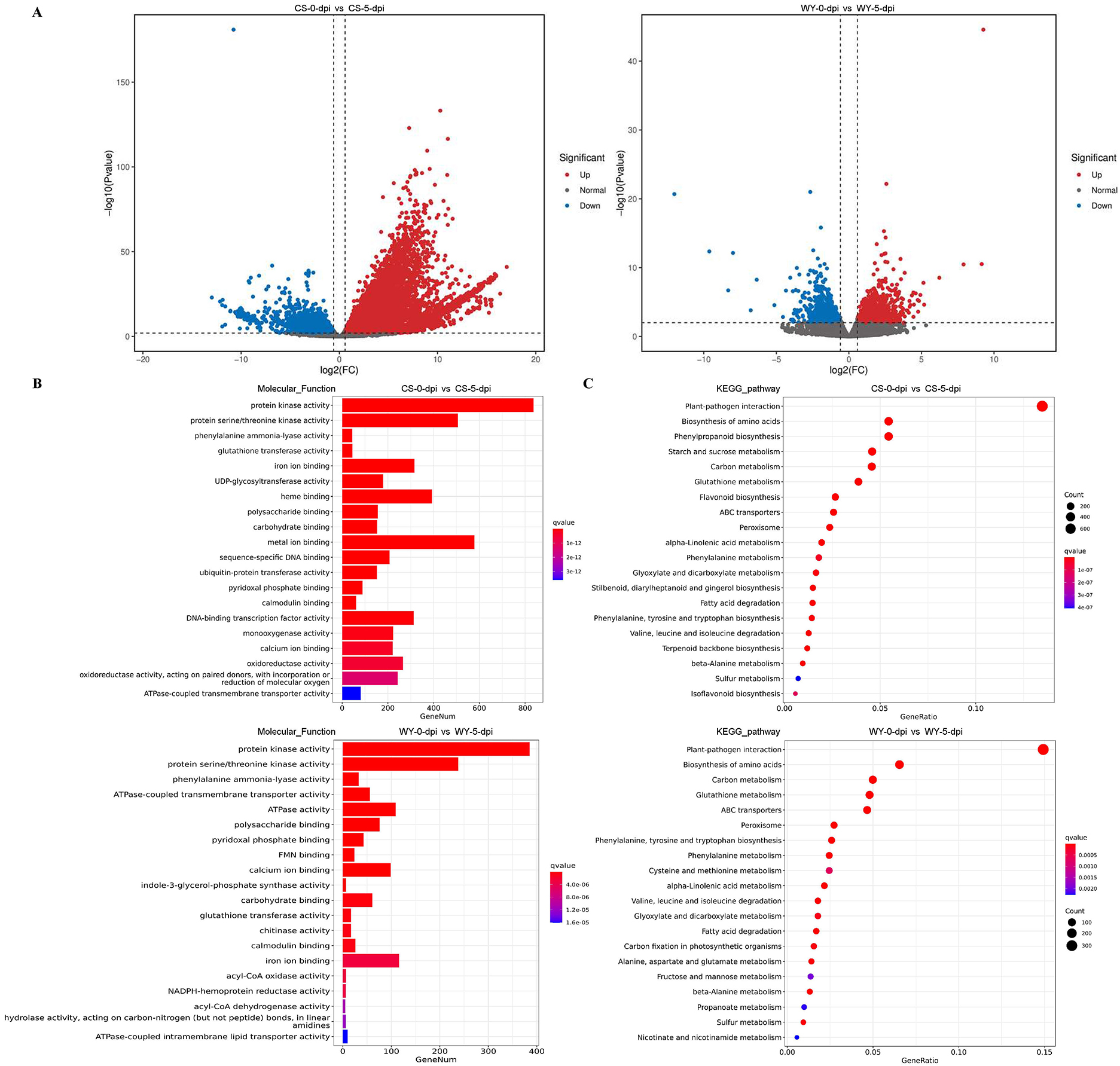
**

**Supplementary Figure 6.** Differential expression analysis for up-regulated genes collected at 5-dpi with *F. graminearum*. (**A**) Volcano plot of DEGs acquired in the comparison of 0 vs. 5-dpi. (**B**) GO term and (**C**) KEGG pathway enrichment analysis for significantly up-regulated DEGs.


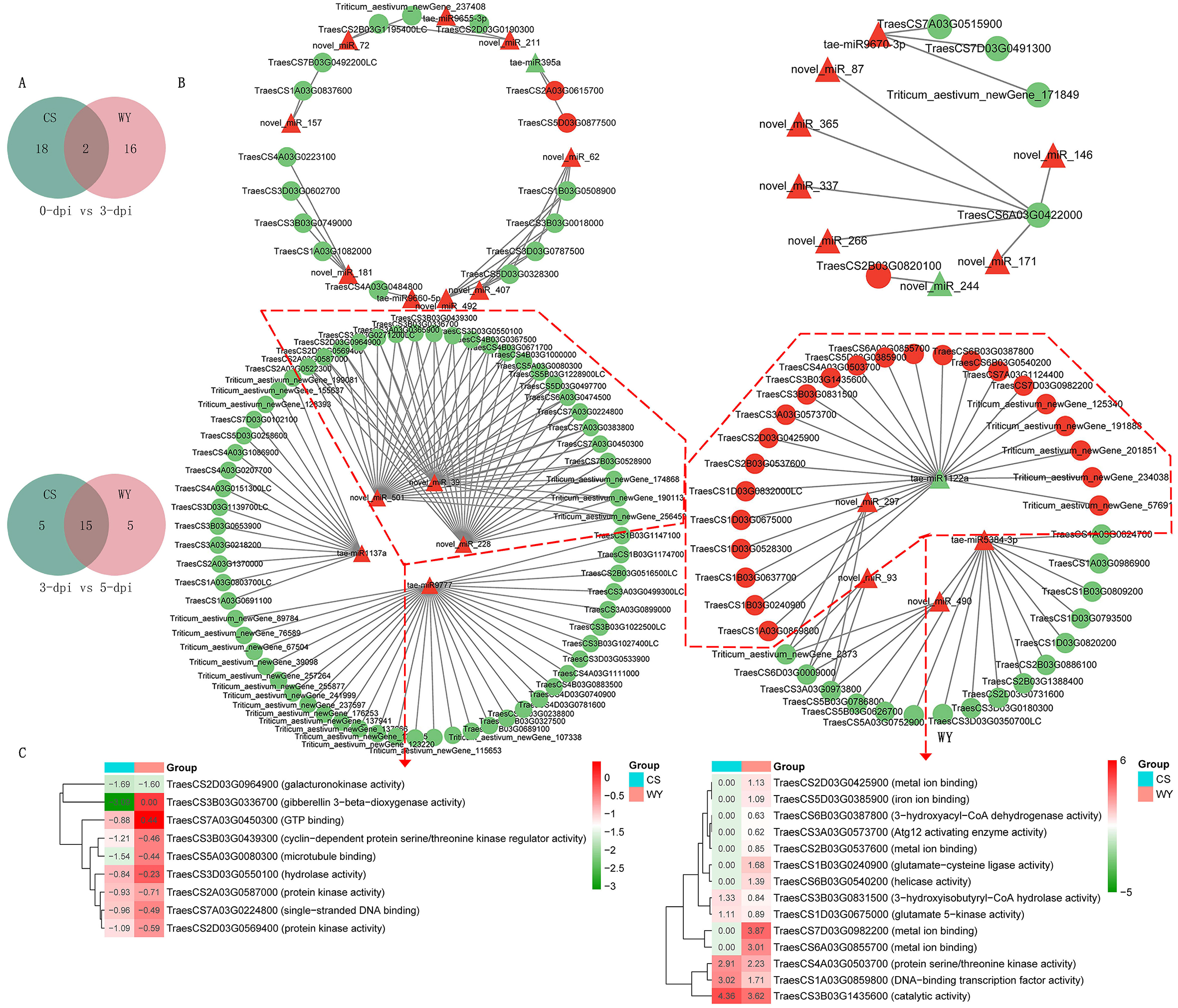


**Supplementary Figure 7.** Targeting relationships between DE_miRNAs and DEGs. (**A**) Comparison of the top 20 DE_miRNAs (or top 18 for 0 vs. 3-dpi in WY) most relevant to target gene regulation in CS and WY. (**B**) Targeting relationships between the DE_miRNAs specifically expressed in CS and WY and DEGs. (**C**) Expression heatmap of genes significantly down-regulated by novel_miR_228 and significantly up-regulated by tae-miR1122a with molecular function (MF) GO term. Heatmap represents log_2_ (FC) between *F. graminearum* infected and control.


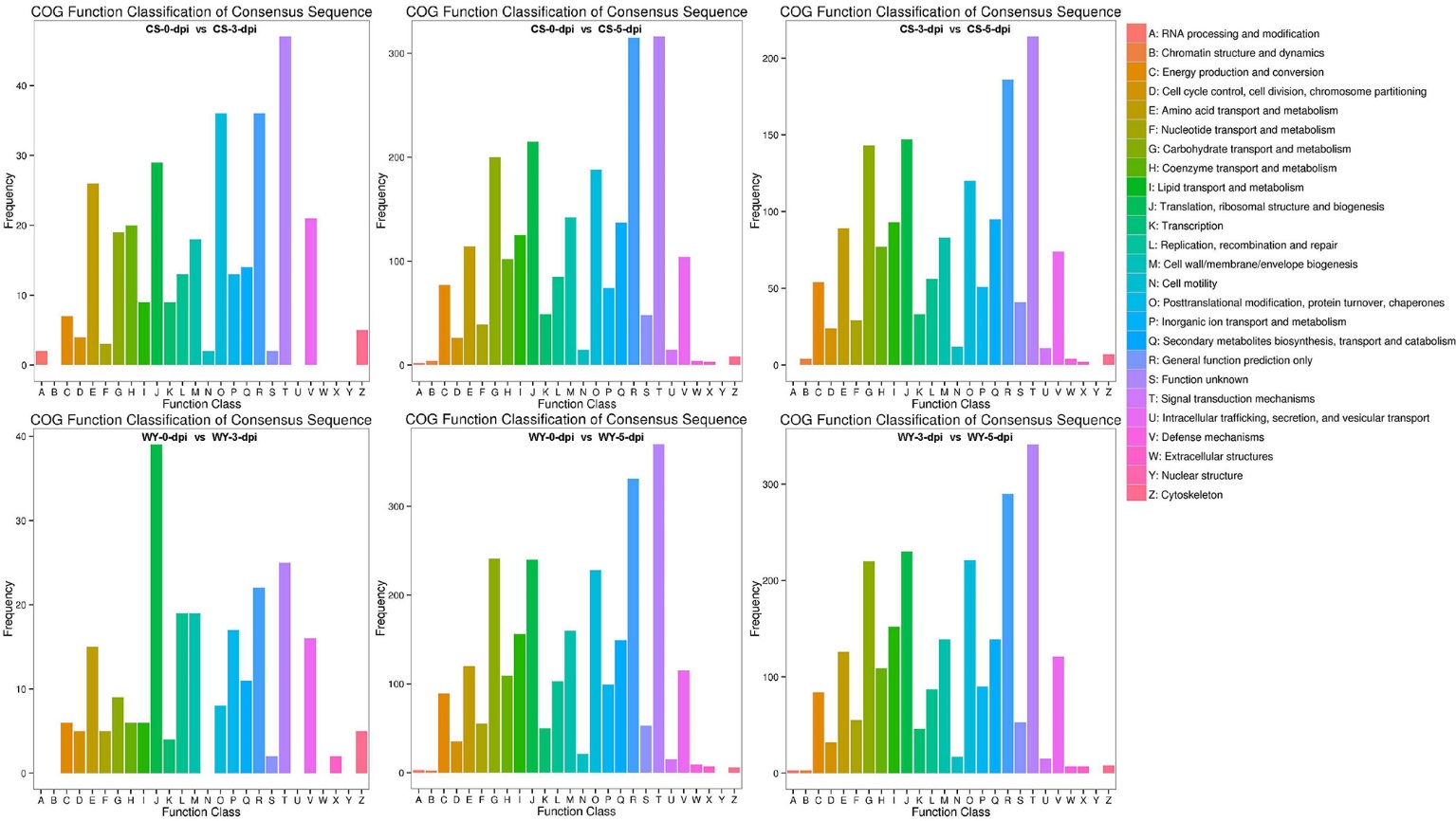


**Supplementary Figure 8.** KOG functional classification of DE_miRNA-regulated DEGs.

**
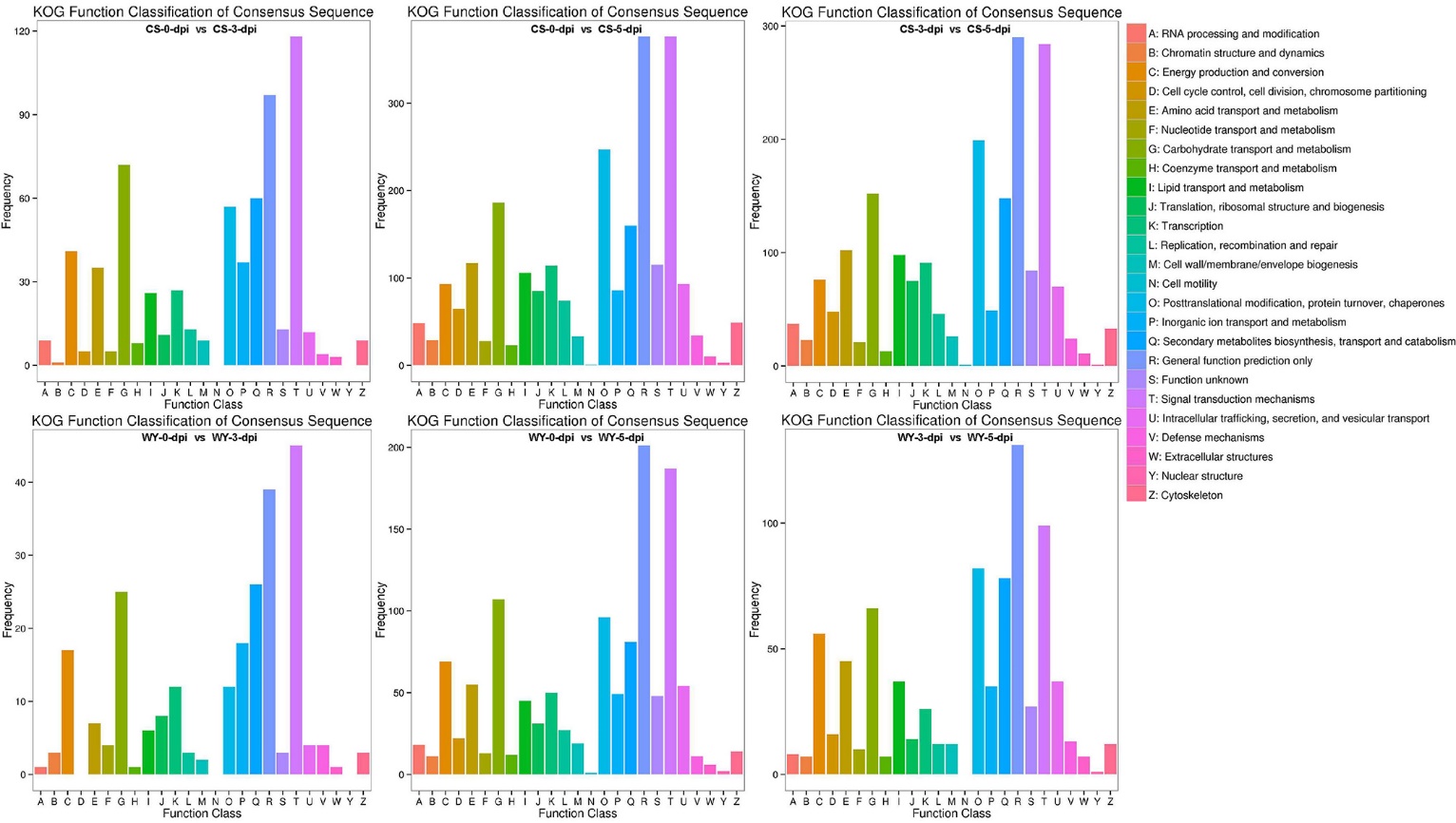
**

**Supplementary Figure 9.** KOG functional classification of miRNA-regulated DEGs.

**
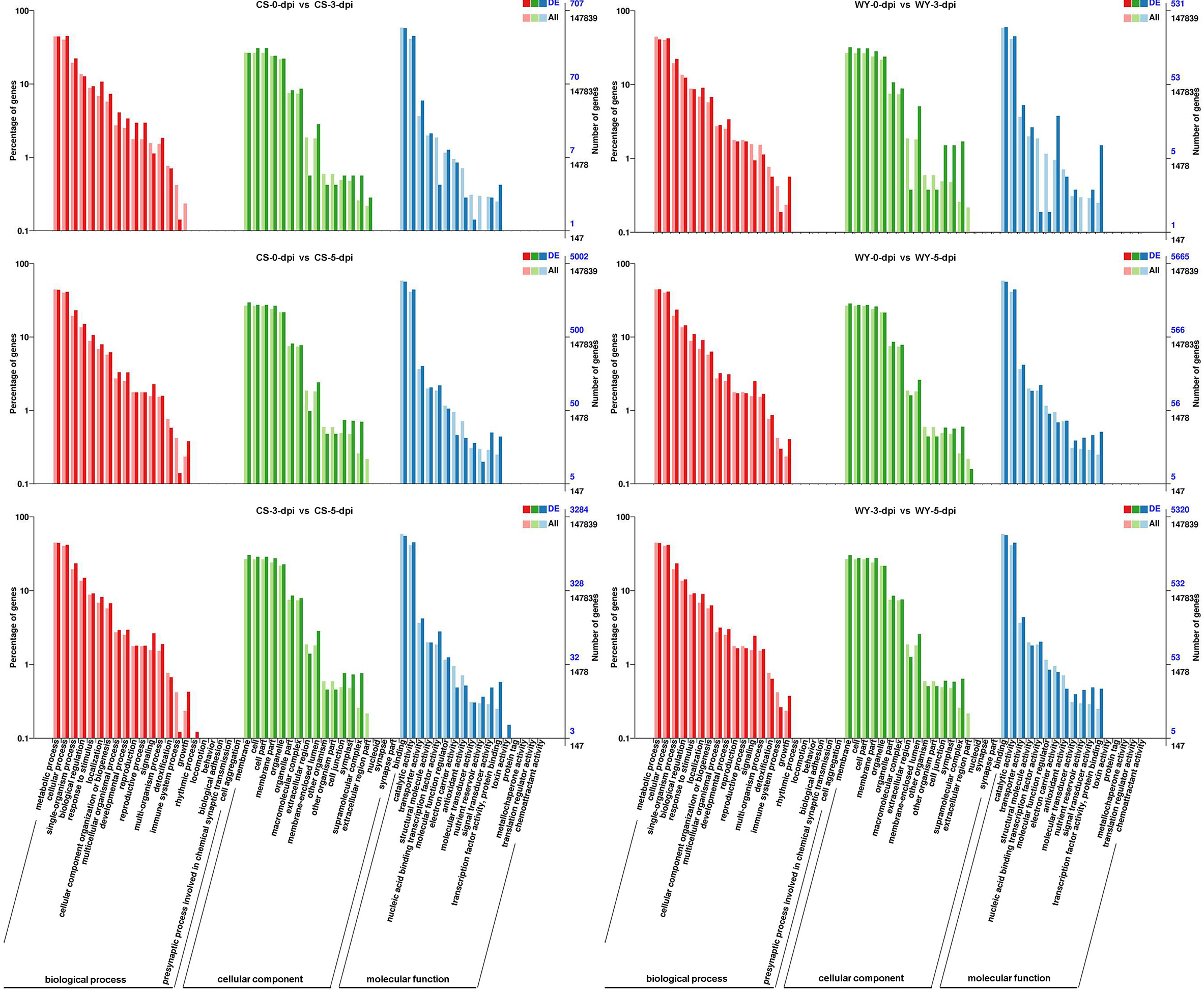
**

**Supplementary Figure 10.** GO classification annotation of DE_miRNA-regulated DEGs.

**
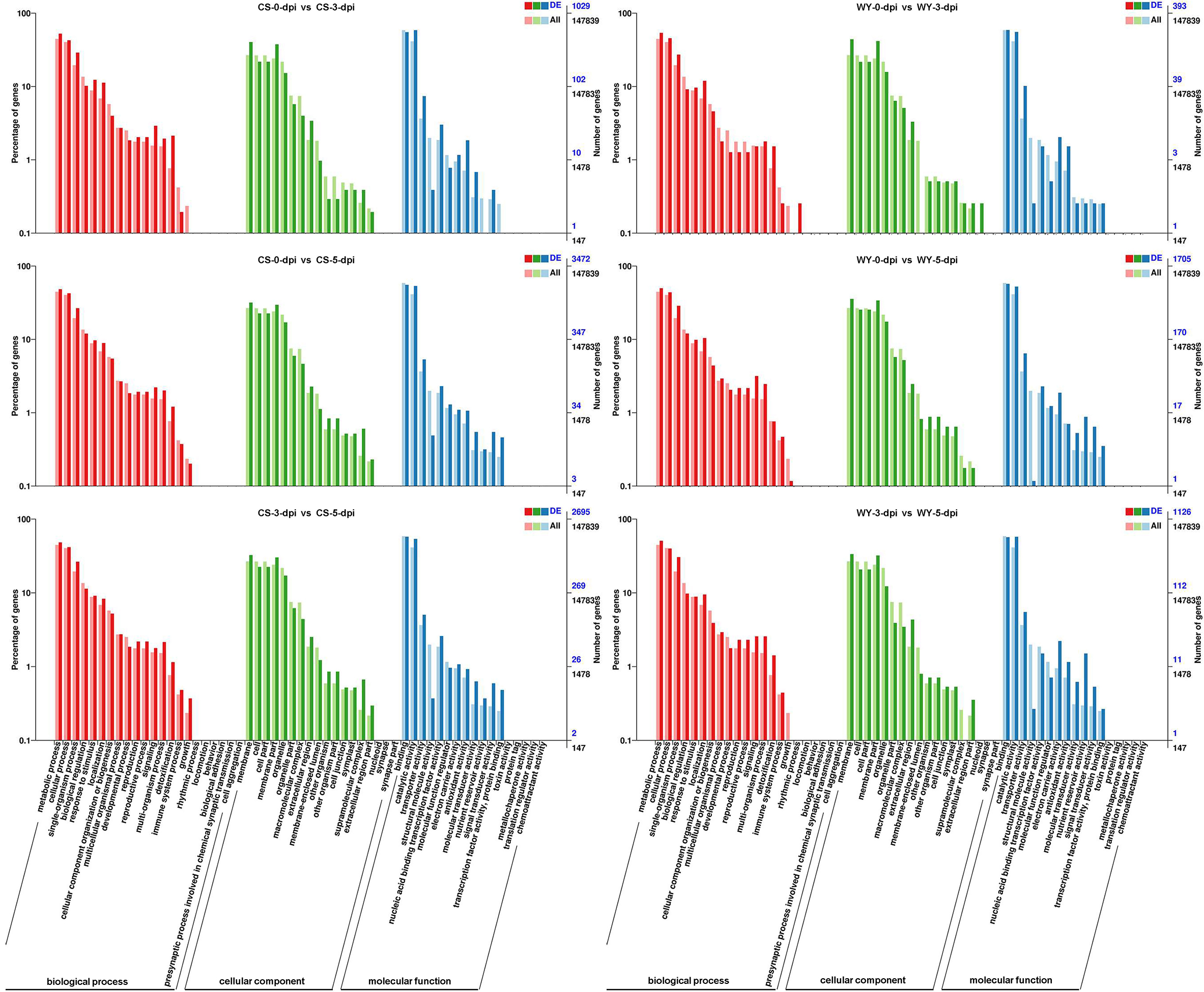
**

**Supplementary Figure 11.** GO classification annotation of miRNA-regulated DEGs.

**
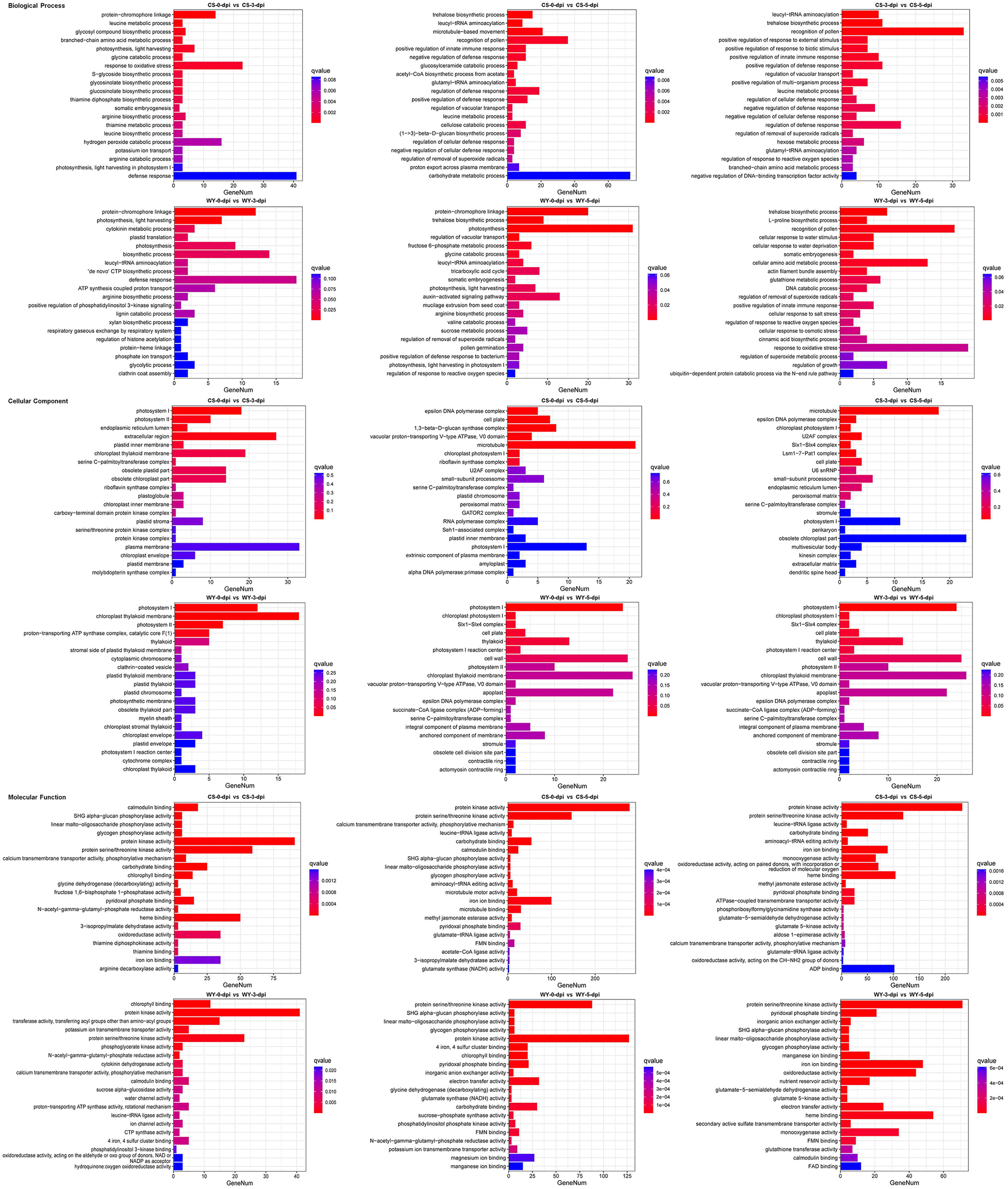
**

**Supplementary Figure 12.** GO term enrichment analysis for miRNA-regulated DEGs. GeneNum indicates the number of genes.

**
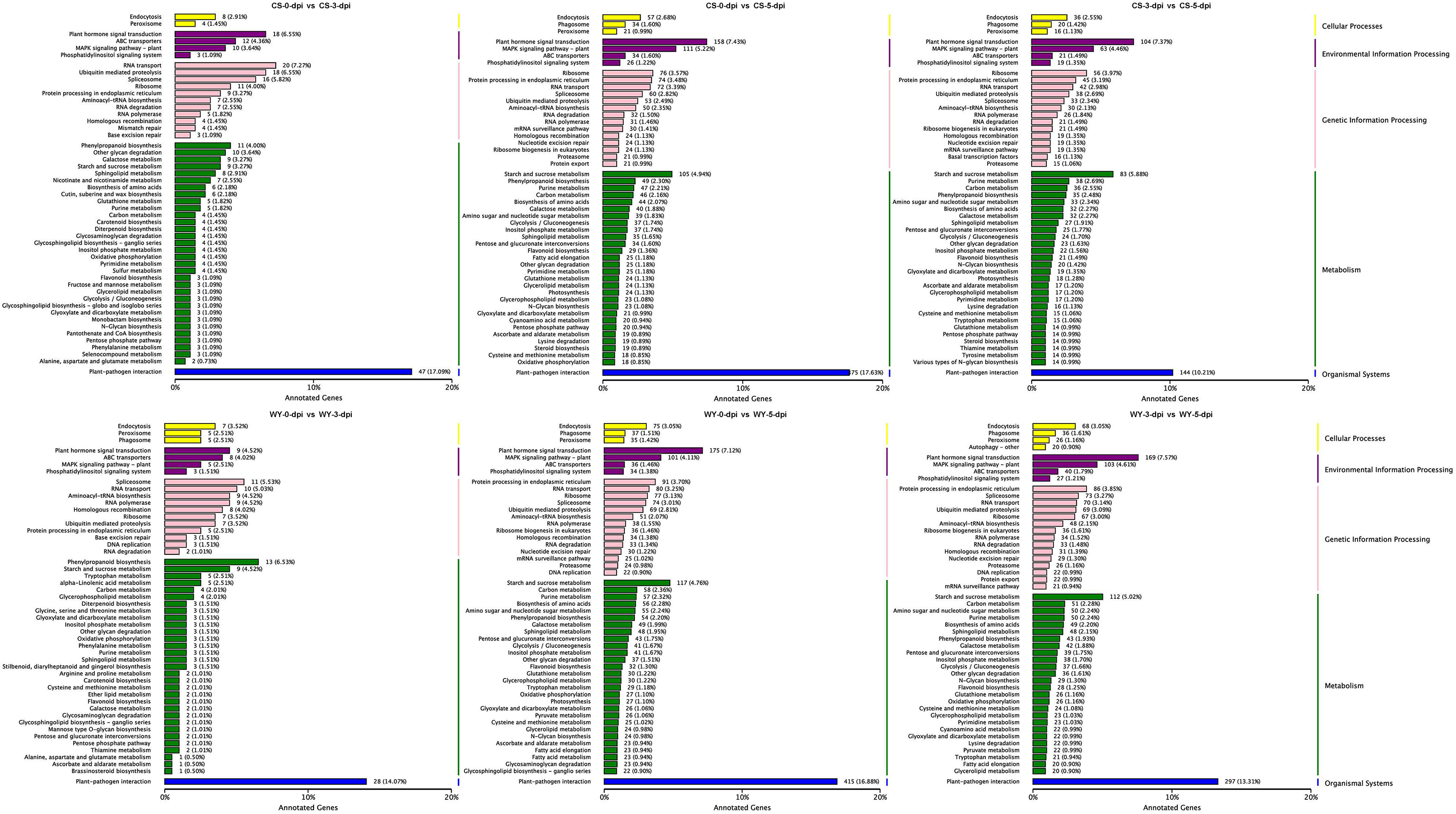
**

**Supplementary Figure 13.** KEGG classification annotation of DE_miRNA-regulated DEGs.

**
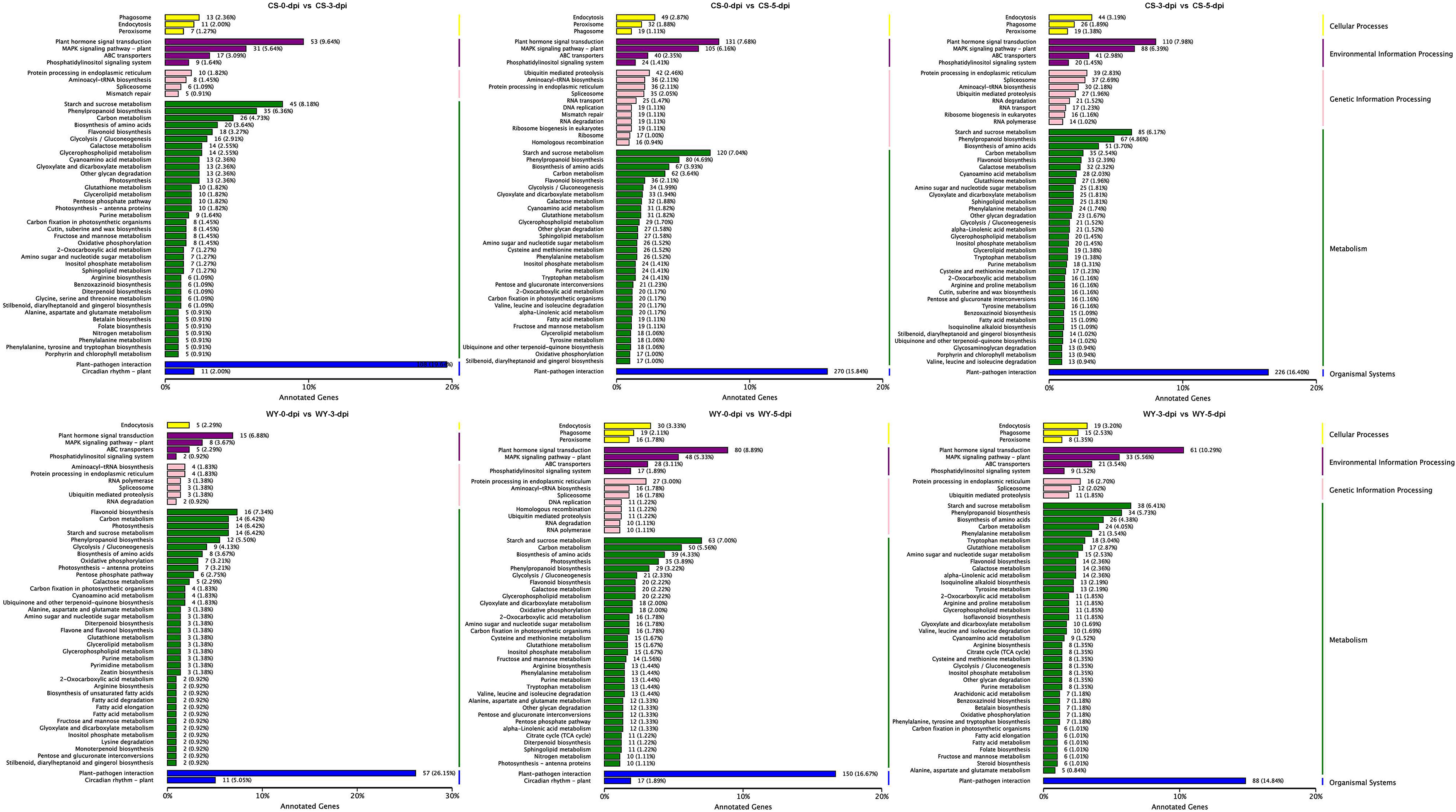
**

**Supplementary Figure 14.** KEGG classification annotation of miRNA-regulated DEGs.

**
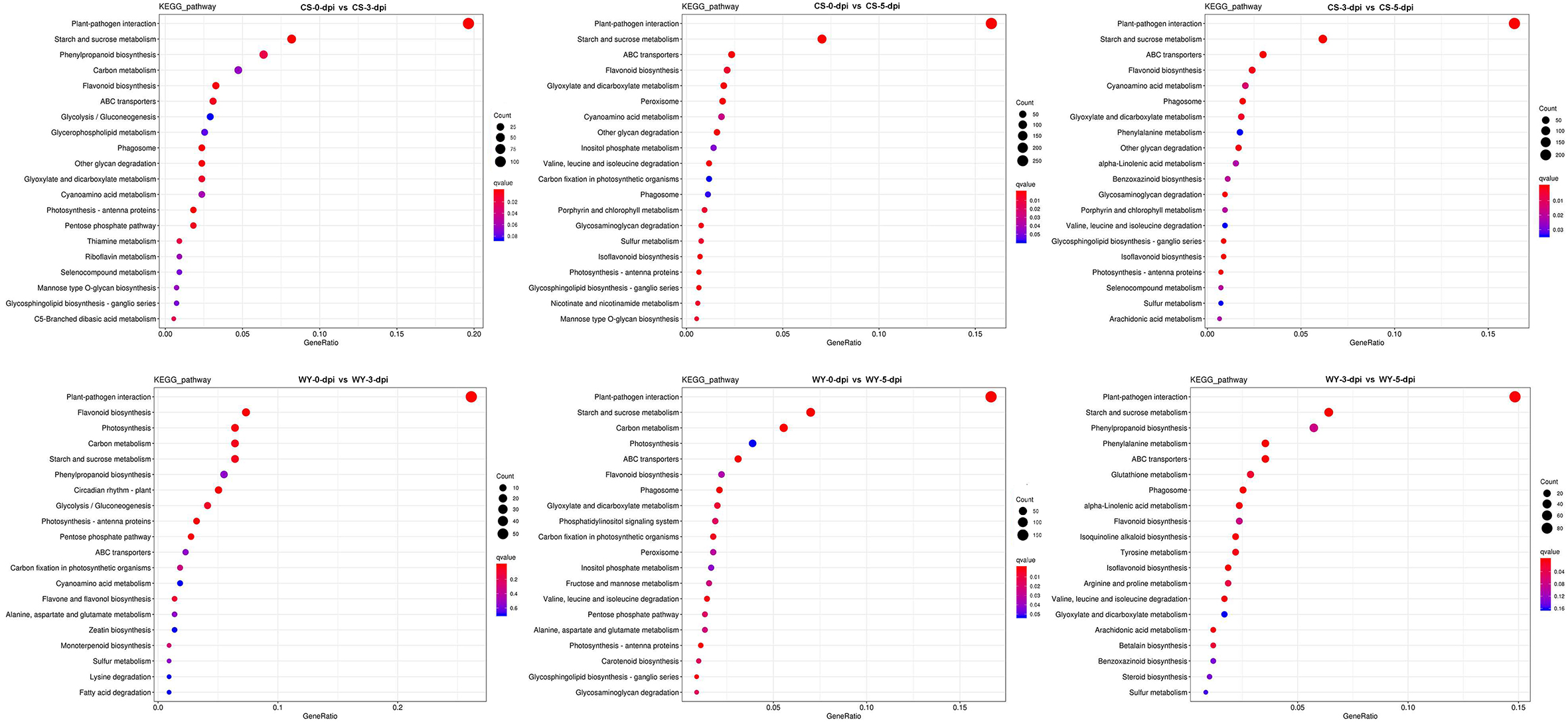
**

**Supplementary Figure 15.** KEGG pathway enrichment analysis for miRNA-regulated DEGs.
